# Tetroazolemycins A and B, Two New Oxazole-Thiazole Siderophores from Deep-Sea *Streptomyces olivaceus* FXJ8.012

**DOI:** 10.3390/md11051524

**Published:** 2013-05-09

**Authors:** Ning Liu, Fei Shang, Lijun Xi, Ying Huang

**Affiliations:** 1 State Key Laboratory of Microbial Resources, Institute of Microbiology, Chinese Academy of Sciences, Beijing 100101, China; E-Mails: fussliu@126.com (N.L.); xilijun1002@163.com (L.X.); 2 Analytical and Testing Center, Beijing University of Chemical Technology, Beijing 100029, China; E-Mail: sjynm220@126.com

**Keywords:** deep-sea water, *Streptomyces olivaceus*, tetroazolemycin, siderophore

## Abstract

Two new oxazole/thiazole derivatives, named tetroazolemycins A (**1**) and B (**2**), have been isolated from the acetone extract of the mycelium of *Streptomyces olivaceus* FXJ8.012 derived from deep-sea water, together with three known compounds, spoxazomicins A–C (**3**–**5**), isolated from the fermentation supernatant. The planar structure and relative configuration of tetroazolemycins were elucidated by a combination of spectroscopic analyses, including 1D- and 2D-NMR techniques, and showed to be new pyochelin-type antibiotics. Both compounds showed metal ion-binding activity and their Zn^2+^ complexes exhibited weak activity against pathogenic bacteria *Klebsiella pneumoniae*.

## 1. Introduction

**Figure 1:**
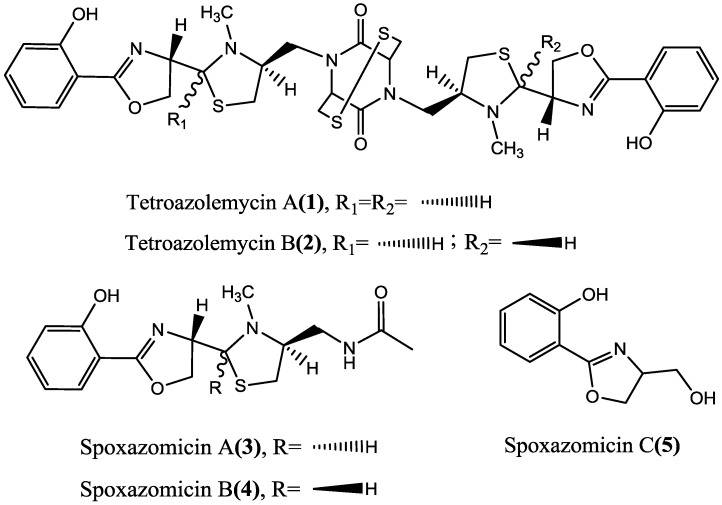
Structures of compounds **1**–**5**.

Actinomycetes, representing one of the most proliﬁc sources for the discovery of bioactive natural products [1,2], have led to the finding of over 50% antibiotics [2] over the past 50 years, such as erythromycin, gentamicin, chloramphenicol, *etc.* The particularity and complexity of marine environments probably endow marine microorganisms the ability to produce different metabolites from those of their terrestrial counterparts [3,4], therefore, the potential for discovering new compounds from marine actinomycetes has been suggested to be far greater than that from terrestrial sources [5,6,7,8]. During our screening program for new natural products from marine actinomycetes, streptomycete strain FXJ8.012, which was isolated from a deep-sea water sample collected from southwest Indian Ocean, was found to produce interesting secondary metabolites besides lobophorins A and B (produced in another culture medium). Further investigation of the metabolites led to the discovery of five siderophores, including two new oxazole/thiazole derivatives (**1**–**2**) and spoxazomicins A–C (**3**–**5**) (Figure 1). Herein we describe the isolation and structural elucidation of the two new compounds, and their activities.

## 2. Results and Discussion

Strain FXJ8.012 formed brown substrate mycelia and abundant grey aerial spore mass after incubation on GYM agar [9] in petri dishes at 28 °C for 7 days. The 16S rRNA gene sequence of this strain was identical with that of *Streptomyces olivaceus* NBRC 12805^T^.

A crude product (1.6 g) was extracted from the mycelia of 12 L fermentation broth of strain FXJ8.012 and was further purified using Sephadex LH-20 column chromatography, preparative thin layer chromatography (PTLC) and preparative high-pressure liquid chromatography (HPLC) to yield compounds **1** (19.1 mg) and **2** (6.6 mg).

### 2.1. Structural Identification of Tetroazolemycins A and B from *Streptomyces olivaceus* FXJ8.012

Tetroazolemycin A (**1**) was obtained as pale yellow solid. Its molecular formula was established as C_34_H_40_N_6_O_6_S_4_ according to the [M + H]^+^ at *m/z* 757.1971 (Calcd for C_34_H_41_N_6_O_6_S_4_, 757.1970) of high resolution-electrospray ionization-mass spectrum (HR-ESI-MS) combined with the ^13^C-nuclear magnetic resonance (NMR) data and corresponding to eighteen degrees of unsaturation. The maxima at 243 and 303 nm in the ultraviolet (UV) spectrum and absorption bands at 1615, 1581, 1491 and 757 cm^−1^ in the infrared (IR) spectra exhibited the presence of *ortho*-substituted phenyl chromophore. Absorption bands at 1668 and 1639 cm^−1^ in the IR spectra also suggested the presence of >C=O ester and >C=N– groups respectively. 

The ^13^C-NMR spectrum of tetroazolemycin A (**1**) showed only half of the expected carbon signals (Table 1), suggesting that this compound had symmetric substructures. The ^1^H-NMR, ^13^C-NMR and 2D-Heteronuclear Single Quantum Coherence (HSQC) spectra revealed the presences of an *ortho*-substituted phenyl (*δ* 6.97, 7.44, 6.92, 7.67 in ^1^H-NMR, bearing a coupling constant *J =* 7.8; *δ* 110.4, 160.1, 116.5, 133.6, 118.6, 128 in ^13^C-NMR), four methylene groups, four non-phenyl methine groups, and one methyl group (Table 1).

**Figure 2:**
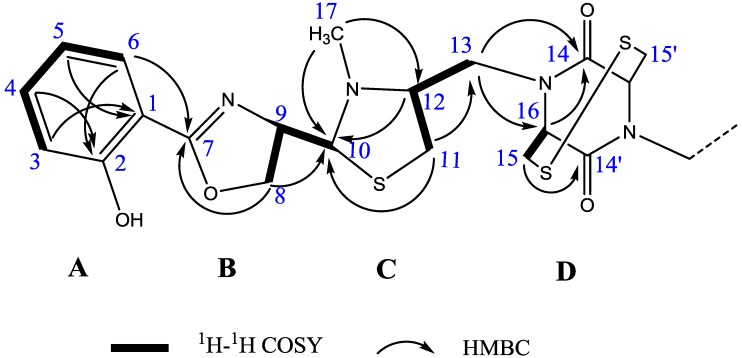
Key ^1^H-^1^H COSY and HMBC correlations of **1**, partial structure.

Detailed analysis of 2D-NMR spectra revealed that **1** was comprised of four ring structures, A–D: a hydroxyphenyl ring (**A**), an oxazoline ring (**B**), a thiazolidine ring (**C**) and a diketopiperazine ring (**D**) (Figure 2). The existence of ring A was previously inferred by UV, IR and 1D-NMR experiments, and was confirmed by ^1^H-^1^H correlation spectroscopy (COSY) and heteronuclear multiple bond correlation (HMBC) experiments: the proton spin system established from H-3 to H-6, the correlations from H-3, H-5 to C-1 and from H-4, H-6 to C-2. By the characteristic carbon signals of C-7 (*δ* 166.2), C-8 (*δ* 69.8) and C-9 (*δ* 71.4), the ^1^H-^1^H COSY correlation of H-8 and H-9, the HMBC correlation of H-8 to C-7, together with the absorption band at 1,639 cm^−1^ in the IR spectra, the substructure of ring B could be constructed as an oxazoline skeleton. The substructure C was deduced as N-methyl thiazolidine ring based on analysis of the characteristic carbon signals of C-10 (*δ* 78.5), C-11 (*δ* 34.2), C-12 (*δ* 70.4), COSY correlation (H-11 and H-12) and HMBC correlations from H-11 and H-12 to C-10. The existence of the N-methyl (*δ*_H_ 2.52 and *δ*_C_ 44.2) on ring C was sustained by the HMBC correlations from H-17 to C-10 and C-12, respectively. The HMBC correlations from H-13 to C-14 and C-16, from H-16 to C-14 and from H-15 to C-14′, together with the chemical shift of C-14 (*δ* 168.8) and ^1^H-^1^H COSY correlation of H-15 and H-16 suggested a diketopiperazine or an azetidine-2-one moiety of ring D. However, the S–S bond could not be reduced using *β*-mercaptoethanol and DTT, and MS/MS of the compounds did not give mass related to azethidine containing monomers (data not shown). Thus, ring D was deduced as a diketopiperazine containing structure.

**Table 1:** ^1^H- (600 MHz) and ^13^C-NMR (150 MHz) Data of **1 **and **2** in Acetone*-d*_6_ (*δ* in ppm, *J* in Hz).

1			2					
Position	*δ*_H_	*δ*_C_, mult.	Position	*δ*_H_	*δ*_C_, mult.	Position	*δ*_H_	*δ*_C_, mult.
1 and 1′	―	110.4, C	1	―	110.4, C	1′	―	110.4, C
2 and 2′	―	160.1, C	2	―	160.0, C	2′	―	160.0, C
3 and 3′	6.97 (d; 7.8)	116.5, CH	3	6.96 (d; 7.8)	116.5, CH	3′	6.96 (d; 7.8)	116.6, CH
4 and 4′	7.44 (dd; 7.8, 7.8)	133.6, CH	4	7.42 (dd; 7.8, 7.8)	133.7, CH	4′	7.42 (dd; 7.8, 7.8)	133.7, CH
5 and 5′	6.92 (dd; 7.8, 7.8)	118.6, CH	5	6.91 (dd; 7.8, 7.8)	118.6, CH	5′	6.91 (dd; 7.8, 7.8)	118.7, CH
6 and 6′	7.67 (d; 7.8)	128, CH	6	7.65 (d; 7.8)	128.0, CH	6′	7.63 (d; 7.8)	128.0, CH
7 and 7′	―	166.2, C=N	7	―	166.2, C=N	7′	―	166.2, C=N
8 and 8′-a	4.47 (m)	69.8, CH2	8-a	4.46 (m)	69.9, CH2	8′-a	4.29 (m)	69.3, CH2
8 and 8′-b	4.62 (m)		8-b	4.62 (m)		8′-b	4.53 (dd; 8.4, 9.6)	
9 and 9′	4.62 (m)	71.4, CH	9	4.61 (m)	71.2, CH	9′	4.77 (m)	67.9, CH
10 and 10′	4.24 (d; 6.0)	78.5, CH	10	4.22 (d; 6.6)	78.6, CH	10′	4.62 (m)	77.1, CH
11 and 11′-a	2.90 (dd; 6.6, 11.4)	34.2, CH2	11-a	2.90 (m)	34.2, CH2	11′-a	2.77 (m)	32.6, CH2
11 and 11′-b	3.15 (dd; 6.6, 11.4)		11-b	3.18 (m)		11′-b	3.00 (m)	
12 and 12′	3.49 (m)	70.4, CH	12	3.49 (m)	70.3, CH	12′	3.84 (m)	67.0, CH
13 and 13′-a	2.92 (dd; 7.2, 13.8)	47.4, CH2	13-a	2.90 (m)	47.5, CH2	13′-a	3.13 (m)	42.6, CH2
13 and 13′-b	4.05 (dd; 6.6, 13.8)		13-b	4.03 (m)		13′-b	4.05 (m)	
14 and 14′	―	168.8, C=O	14	―	168.9, C=O	14′	―	169.3, C=O
15 and 15′	3.65 (d; 3.6)	45.4, CH2	15	3.64 (m)	45.4, CH2	15′	3.62 (m)	45.6, CH2
16 and 16′	4.75 (t; 3.8)	60.1, CH	16	4.78 (m)	60.2, CH	16′	4.75 (m)	59.4, CH
17 and 17′	2.52 (s)	44.2, CH3	17	2.54 (s)	44.4, CH3	17′	2.45 (s)	36.2, CH3

The connectivities of substructures A–D were elucidated by the HMBC and ^1^H-^1^H COSY experiments (Figure 2). The connectivity between ring A and ring B was confirmed by the long-range coupling from H-6 to C-7. The HMBC correlation from H-8 to C-10 and COSY correlation of H-9 and H-10 established the linkage between ring B and ring C. The HMBC correlation from H-11 to C-13, from H-13 to C-14 and C-16, together with the COSY correlation of H-11/H-12/H-13, established the linkage between ring C and ring D. Considering the molecular formula, the remaining sulfur atom of **1** should be assigned to C-15 and C-15′ to form a S–S bond.

**Figure 3:**
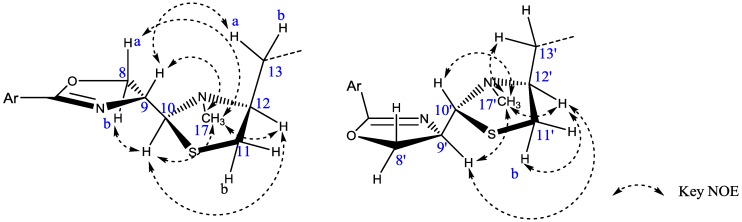
Key NOE correlations of half structures **of 1** (left) and **2** (right).

In the NOE difference experiments, an irradiation of H-10 resulted in the enhancement of H-12 and H-17, indicating that H-10 and H-12 were on one face of ring C and that H-9 and H-13 were on the other face. This deduction was further sustained by the correlations between H-9 and H-13a in a 2D-NOESY experiment (Figure 3, left). Irradiations of H-8a and H-8b both resulted in the enhancement of H-17, indicating that H-8 and H-17 were on the same side of the paper plane. Other Key NOE correlations were shown in Figure 3. Therefore, the relative configurations of C-9, C-10 and C-12 of compound **1** were proposed to be 9R*, 10S*, and 12S*. This proposal was sustained by the co-production of spoxazomicin A from a biogenetic perspective.

Tetroazolemycin B (**2**) was also obtained as pale yellow solid, and its molecular formula was established as C_34_H_40_N_6_O_6_S_4_ according to the [M + H]^+^ at *m/z* 757.1965 (Calcd for C_34_H_41_N_6_O_6_S_4_, 757.1970) in HR-ESI-MS combined with the ^13^C-NMR data and corresponding to eighteen degrees of unsaturation, too. It’s UV, IR, ^1^H- and ^13^C-NMR spectra were similar to those of tetroazolemycin A (**1**). Careful analysis of its ^1^H-NMR, ^13^C-NMR and 2D-NMR data revealed that half structure of this compound was identical with that of **1**, and the planar structure of the other half was still in accordance with **1**. Differences lied in the chemical shifts of carbons 8′ to 13′ and 17′, implying a different relative configuration of this half structure. Further 2D-NOESY revealed the correlation between H-9′ and H-12′ (Figure 3, right), suggesting that H-10′ and H-12′ were not on the same side of ring C. No correlation was found between H-8′ and H-17′ in 2D-NOESY experiment; and in the NOE difference experiment, neither the irradiation of H-8′ nor H-8′b resulted in the enhancement of H-17′, indicating that H-8′ and H-17′ were on the opposite side of the paper plane. Other Key NOE correlations were shown in Figure 3. Considering the co-production of spoxazomicin B and from a biogenetic perspective, the relative configurations of C-9, C-10 and C-12 of compound **2** were suggested as 9R*, 10S*, 12S*, 9′R*, 10′R*, and 12′S*.

### 2.2. The Activities of Compounds 1 and 2

Tetroazolemycins A (**1**) and B (**2**) were evaluated for their heavy metal ion-binding ability. When Fe^3+^, Cu^2+^ and Zn^2+^ solutions were added into tetroazolemycins A (**1**) and B (**2**)-acetone solutions, mauve, blue and white precipitates could be observed respectively and immediately, while no precipitation or color change occurred when Pb^2+^, Cr^3+^ or Mn^2+^ solutions were added. In reverse, when tetroazolemycin A (**1**) and B (**2**)-acetone solutions were added into the metal ion solutions, brown, blue and white precipitates could be observed immediately in Fe^3+^, Cu^2+^ and Zn^2+^ solutions respectively, while no precipitates or color change occurred in Pb^2+^, Cr^3+^ or Mn^2+^ solutions. These facts indicated that tetroazolemycins A (**1**) and B (**2**) had affinity for Fe^3+^, Cu^2+^ and Zn^2+^ but not for Cr^3+^, Mn^2+^ or Pb^2+^.

The compounds and their metal ion complexes turned out to be inactive against microbes *Staphyloccocus aureus*, *Escherichia coli*, *Bacillus subtilis*, *Mycobacterium gilvum*, *Pseudomonas aeruginosa*, *Candida albicans*, *Candida pseudorugosa*, *Aspergillus fumigatus*, *Fusarium oxysporum* and *Rhizoctonia solani*, inactive against P388D and A549 tumor cell lines, and inactive against influenza A H1N1 (A/WSN/33) virus. Although tetroazolemycins A (**1**) and B (**2**) showed no activity against *Klebsiella pneumoniae* either, their Zn^2+^ complexes weakly inhibited this pathogen with MICs of 125–250 µg/mL and 125 µg/mL, respectively.

### 2.3. Discussion

Tetroazolemycins A (**1**) and B (**2**) are structurally classified as oxazole/thiazole siderophores with high affinity for ferric ion [10]. A large number of siderophores with different types of structures have been isolated so far from microorganisms [11]. The chemical structures of **1** and **2** are closely related to those of spoxazomicins [12], pyochelin [13], thiazostatin [14], wastemycins [15], and transvalencins [16]. However, this is the first report of homo/hetero-dimer siderophores of pyochelin family antibiotics. It is hard to determine the absolute configurations for pyochelin-type siderophores due to their multi-chiral centers and isomerization on ring C, so most of the pyochelin-type siderophores, including spoxazomicins, were reported without absolute stereochemistry. We have not resolved the absolute configurations of tetroazolemycins either, for the structures contain neither available hydroxyl groups for Mosher’s reaction nor available amino acids for Marfey’s reaction, no CD spectra from reference compounds have been reported, and our effort for crystallizing tetroazolemycins and their metal ion complexes was failed. 

Tetroazolemycins A (**1**) and B (**2**) are functionally unique owing to their binding ability to Fe^3+^, Cu^2+^ and Zn^2+^, like desferrioxamine E, which was also reported to be able to bind multiple metal ions besides Fe^3+^ [17]. Because the antimicrobial activity is not exhibited by the free-state tetroazolemycins but by their Zn^2+^-complexes, like transvalencin A, and because the gene cluster of coelibactin, a putative pyochelin-type siderophore, was regulated by Zn^2+^ [18], we suppose that the oxazole/thiazole siderophores may be closely related to Zn^2+^ besides Fe^3+^, and the *in situ* function of them probably needs to be re-evaluated. On the other hand, tetroazolemycins A (**1**) and B (**2**) are sulfur-containing compounds. As many sulfur-containing natural products, such as bleomycin, cyclothiazomycin, diphenazithionin, penicillin, *etc.*, exhibit significant bioactivity, we speculate that tetroazolemycins possess potential bioactivities although no obvious antibiotic activity was detected in this study. Further studies on the absolute configuration and more pharmacological functions of tetroazolemycins are in progress.

## 3. Experimental Section

### 3.1. General

UV spectra were recorded on a Beckman Coulter DU800 UV/Vis Spectrophotometer, 190 to 800 nm. IR spectra were recorded on a Thermo Nicoiet 8,700 spectrometer. Optical rotation was run on an ATAGO POLAX-2L polarimeter. ESI-MS spectra were performed on a Thermo-Finnigan LCQ DECA XP mass spectrometer. HR-ESI-MS spectra were obtained on a Waters Xevo G2 QTOF mass spectrometer. NMR spectra were measured on a Bruker AV 600/400 NMR spectrometer. The concentration was performed on an EYELA N-1100S-W rotary evaporator. The analytical and preparative HPLC were both performed on a Shimadzu SPD-M20A HPLC system with a Waters Xbridge ODS (10 × 150 mm, 5 μm) column and a Sigma-aldrich Ascentis RP-Amide column (4.6 × 150 mm, 5 μm). Column chromatography was carried out on silica gel (100–200 mesh) (Qingdao Haiyang Chemical Group Corp., Qingdao, China) and Sephadex LH-20 (Pharmacia, Uppsala, Sweden). Thin layer chromatography (TLC) was carried out on silica gel GF254 plate (1.05554.0001, MERCK, Darmstadt, Germany). PTLC was carried out on silica gel GF254 preparative plate (Qingdao Haiyang Chemical Group Corp., Qingdao, China).

### 3.2. Actinomycete Strain

*Streptomyce* *olivaceus* FXJ8.012 was isolated from a deep-sea water sample collected from southwest Indian Ocean (S37.83°, E72.00°) at a depth of 3,838 m, during the DY115-20 cruise of DaYang YiHao research vessel in 2009. The isolation and growth media were both GYM agar. The strain was identified by morphology and 16S rRNA gene sequence analysis using regular procedures [19], and was deposited in the China General Microbiological Culture Collection Centre (CGMCC), Beijing, China, under the accession number CGMCC 4950. 

### 3.3. Fermentation, Extraction and Isolation

After growth on GYM agar in petri dishes at 28 °C for 7 days, suitable amount of spores of *Streptomyces olivaceus* FXJ8.012 were transferred to a 500-mL shake ﬂask containing 100 mL of liquid GYM medium and incubated on a rotary shaker at 170–180 rpm and 28 °C for 5 days as the seed culture. The seed culture was used to inoculate 120 bottles of 500-mL shake ﬂasks each containing 100 mL of SGG medium [20], followed by incubation under similar conditions for 7 days. 

After fermentation, the culture broth (12 L) was centrifugated at 5000 rpm for 15 min to separate the mycelia and supernatant. The resulting mycelium cake was extracted three times with equal volume acetone for three hours. The acetone-extracted portion was then concentrated *in vacuo* to evaporate the solvent, and subjected to silica gel (100–200 mesh) column chromatography using a gradient of CHCl_3_/MeOH (100%–90%) to obtain the crude product (1.6 g). The crude product was subjected to Sephadex LH-20 (MeOH) to get rid of fatty acids, nucleosides and other impurities. PTLC (20 × 20 cm, CHCl_3_:MeOH = 10:1.2) was followed to obtain fraction I (41 mg). Final purification was carried out sequentially by the RP-HPLC with Waters Xbridge ODS column (MeOH:H_2_O = 70:30, v/v) and Sigma-aldrich Ascentis RP-Amide column (MeOH:H_2_O = 64:36, v/v) to yield **1 **(19.1 mg) and **2 **(6.6 mg).

After concentrated *in vacuo* to about 3 L, the fermentation supernatant was extracted three times with equal volume ethyl acetate. The ethyl acetate portion was then concentrated *in vacuo* to evaporate the solvent, and subjected to silica gel (100–200 mesh) column chromatography using a gradient of CHCl_3_/MeOH (100%–90%) to obtain the crude product (0.5 g). The crude product was subjected to Sephadex LH-20 (MeOH), and further purified sequentially by the RP-HPLC with Waters Xbridge ODS column (MeOH:H_2_O = 80:20, v/v) and Sigma-aldrich Ascentis RP-Amide column (Acetonitrile:H_2_O = 65:35, v/v) to yield **3 **(3.3 mg), **4** (1.3 mg) and **5 **(0.7 mg).

Tetroazolemycin A (**1**): pale yellow solid; [α]^25^_D_ (*c* 0.1, MeOH) = −10.7°; UV (MeOH, *c* = 0.05) λ_max_ (log ε) 242 (1.13), 304 (0.88) nm; IR (KBr) ν_max_ 3431, 3060, 2926, 2856, 1668, 1639, 1615, 1581, 1491, 1460, 1367, 1258, 1231, 757.1 cm^−1^; ^1^H- and ^13^C-NMR data, Table 1; ESI-MS *m/z* 757 [M + H]^+^, 779 [M + Na]^+^; HR-ESI-MS *m/z* 757.1971 [M + H]^+^ (Calcd for C_34_H_41_N_6_O_6_S_4_, 757.1970) (see supplementary Figures S1–S13).

Tetroazolemycin B (**2**): pale yellow solid; [α]^25^_D_ (*c* 0.03, MeOH) = −17.4°; UV (MeOH, *c* = 0.05) λ_max_ (log ε) 241 (1.11), 304 (0.84) nm; IR (KBr) ν_max_ 3434, 3064, 2926, 2858, 1669, 1640, 1615, 1581, 1491, 1460, 1367, 1258, 1230, 758 cm^−1^; ^1^H- and ^13^C-NMR data, Table 1; ESI-MS *m/z* 757 [M + H]^+^, 779 [M + Na]^+^; HR-ESI-MS *m/z* 757.1965 [M + H]^+^ (Calcd for C_34_H_41_N_6_O_6_S_4_, 757.1970) (see supplementary Figures S14–S23).

Spoxazomicin A (**3**): yellow solid; UV (MeOH:H_2_O 70:30) 242, 301 nm; ^1^H- and ^13^C-NMR data, supplementary Table S1; HR-ESI-MS *m/z* 336.1384 [M + H]^+^ (Calcd for C_16_H_21_N_3_O_3_S, 336.1382) (see supplementary Figures S24–S26).

Spoxazomicin B (**4**): yellow solid; UV (MeOH:H_2_O 70:30) 242, 301 nm; ^1^H- and ^13^C-NMR data, Table S1. HR-ESI-MS *m/z* 336.1385 [M + H]^+^ (Calcd for C_16_H_21_N_3_O_3_S, 336.1382) (see supplementary Figures S27–S29).

Spoxazomicin C (**5**): colorless needles; UV (MeOH:H_2_O 70:30) 242, 301 nm; ^1^H-NMR data, Table S1. HR-ESI-MS *m/z* 194.0824 [M + H]^+^ (Calcd for C_10_H_11_NO_3_, 194.0817) (see supplementary Figures S30 and S31).

### 3.4. Metal Ion Binding Assay

0.1 M solutions of FeCl_3_, CuSO_4_, ZnCl_2_, PbCl_2_, CrCl_3_ and MnCl_2_ were prepared fresh. Several microliters of each solution were added into 0.5–1 mL tetroazolemycins A (**1**) and B (**2**)-acetone solutions respectively, and vice versa. Metal ion binding ability was determined by observing the precipitation of the complexes and color change of the solutions.

### 3.5. Bioactivity Assay

Primary antimicrobial assays were conducted using agar-diffusion method: 50 µg compound solution was transferred to a sterile filter paper disc of 5 mm diameter, and put onto the surface of LB agar [21] or PDA agar [22] containing pathogenic bacteria or fungi, followed by incubation at 37 °C overnight for bacteria or 25 °C for 1–2 days for fungi. Bioactivity was determined by observing the inhibition zone. MIC assays were conducted in triplicate using liquid cultures in 96-well culture plates according to a modified method described by Shapiro [23].

Antitumor activity was tested against murine macrophage cell line P388D and human lung adenocarcinoma cell line A549, using MTT method as described previously [24]. The result of anti-influenza A H1N1 (A/WSN/33) virus was provided by professor Xin Ye at the Institute of Microbiology, Chinese Academy of Sciences (IMCAS).

## 4. Conclusions

Five oxazole/thiazole siderophores **1**–**5** were isolated from marine *Streptomyces olivaceus* FXJ8.012 and their structures were elucidated. The major compounds tetroazolemycins A (**1**) and B (**2**) represent novel chemical structures and are the first discovered dimer forms of pyochelin-type siderophores. Tetroazolemycins showed affinity for Fe^3+^, Cu^2+^ and Zn^2+^, and the Zn^2+^ complexes of **1** and **2** weakly inhibited pathogen *Klebsiella pneumoniae*.

## Supplementary Files

Supplementary File 1 Supplementary (PDF, 807 KB)
