# Supplementary material for: Tetroazolemycins A and B, Two New Oxazole-Thiazole Siderophores from Deep-Sea Streptomyces olivaceus FXJ8.012
Source: Mar Drugs. 2013 May 9;11(5):1524–33. doi: 10.3390/md11051524 (PMC3707159; doi:10.3390/md11051524)

Supplementary Information

Table S1. ^{1}H and ^{13}C-NMR Data of **3**–**5** (δ in ppm, J in Hz).
Figure S1. HR-ESI-MS spectrum of **1**.
Figure S2. UV spectrum of **1**.
Figure S3. IR spectrum of **1**.
Figure S4. ^{1}H-NMR spectrum (600 MHz, Acetone-d_6) of **1**.
Figure S5. ^{13}C-NMR spectrum (150 MHz, Acetone-d_6) of **1**.
Figure S6. DEPT 135 spectrum (150 MHz, Acetone-d_6) of **1**.
Figure S7. ^{1}H–^{1}H-COSY spectrum (600 × 600 MHz, Acetone-d_6) of **1**.
Figure S8. ^{1}H–^{13}C-HSQC spectrum (600 × 150 MHz, Acetone-d_6) of **1**.
Figure S9. ^{1}H–^{13}C-HMBC spectrum (600 × 150 MHz, Acetone-d_6) of **1**.
Figure S10. ^{1}H–^{1}H-ROESY spectrum (600 × 600 MHz, Acetone-d_6) of **1**.
Figure S11. NOE difference spectrum (H-10 irradiated) (600 MHz, Acetone-d_6) of **1**.
Figure S12. NOE difference spectrum (H-8a irradiated) (600 MHz, Acetone-d_6) of **1**.
Figure S13. NOE difference spectrum (H-8b irradiated) (600 MHz, Acetone-d_6) of **1**.
Figure S14. HR-ESI-MS spectrum of **2**.
Figure S15. UV spectrum of **2**.
Figure S16. IR spectrum of **2**.
Figure S17. ^{1}H-NMR spectrum (600 MHz, Acetone-d_6) of **2**.
Figure S18. ^{13}C-NMR spectrum (150 MHz, Acetone-d_6) of **2**.
Figure S19. DEPT 135 spectrum (150 MHz, Acetone-d_6) of **2**.
Figure S20. ^{1}H–^{1}H-COSY spectrum (600 × 600 MHz, Acetone-d_6) of **2**.
Figure S21. ^{1}H–^{13}C-HSQC spectrum (600 × 150 MHz, Acetone-d_6) of **2**.
Figure S22. ^{1}H–^{13}C-HMBC spectrum (600 × 150 MHz, Acetone-d_6) of **2**.
Figure S23. ^{1}H–^{1}H-ROESY spectrum (600 × 600 MHz, Acetone-d_6) of **2**.
Figure S24. HR-ESI-MS spectrum of **3**.
Figure S25. ^{1}H-NMR spectrum (600 MHz, CD_3OD) of **3**.
Figure S26. ^{13}C-NMR spectrum (150 MHz, CD_3OD) of **3**.
Figure S27. HR-ESI-MS spectrum of **4**.
Figure S28. ^{1}H-NMR spectrum (600 MHz, CD_3OD) of **4**.
Figure S29. ^{13}C-NMR spectrum (150 MHz, CD_3OD) of **4**.
Figure S30. HR-ESI-MS spectrum of **5**.
Figure S31. ^{1}H-NMR spectrum (400 MHz, Acetone-d_6) of **5**.

Table S1. ^{1}H and ^{13}C-NMR Data of **3**–**5** (δ in ppm, *J* in Hz).

	Spoxazomicin A (3)		Spoxazomicin B (4)		Spoxazomicin C (5)
**Position**	δ_C	δ_H	δ_C	δ_H	δ_H
1	110.3	—	110.4	—	
2	159.7	—	159.6	—	
3	116.1	6.95 (d; 8.4)	116.1	6.95 (d; 8.4)	6.93 (d; 8.0)
4	133.2	7.39 (dd; 8.4,7.8)	133.2	7.40 (dd; 8.4,7.8)	7.41 (dd; 8.0,8.0)
5	118.4	6.89 (dd; 7.8,7.8)	118.4	6.89 (dd; 7.8,7.8)	6.88 (dd; 7.6,7.6)
6	127.7	7.66 (d; 7.8)	127.8	7.66 (d; 7.8)	7.63 (d; 7.6)
7	166.3	—	166.3	—	
8	69.4	4.42 (dd; 7.2,8.4)	69.2	4.3 (dd; 8.4,8.4)	4.4 (m)
		4.54 (dd; 8.4,9.6)		4.51 (dd; 9.0,9.0)	4.51 (m)
9	71.3	4.65 (m)	68.3	4.73 (m)	4.44 (m)
10	76.5	4.20 (d; 5.4)	77	4.49 (d; 7.2)	3.72 (m)
11	33.3	2.87 (dd; 8.4,10.8)	31.9	2.74 (dd; 5.4,10.2)	
		2.99 (dd; 5.4,10.8)		3.04 (dd; 5.4,10.2)	
12	71.4	3.13 (m)	67.7	3.58 (m)	
13	41.2	3.21 (dd; 6.6,13.8)	38	3.36 (dd; 7.8,13.8)	
		3.48 (dd; 4.2,13.8)		3.48 (dd; 7.8,13.8)	
14	172.1	—	172	—	
15	21.2	1.99 (s)	21.1	1.95 (s)	
16	41.6	2.52 (s)	35.4	2.50 (s)	

Figure S1. HR-ESI-MS spectrum of **1**.

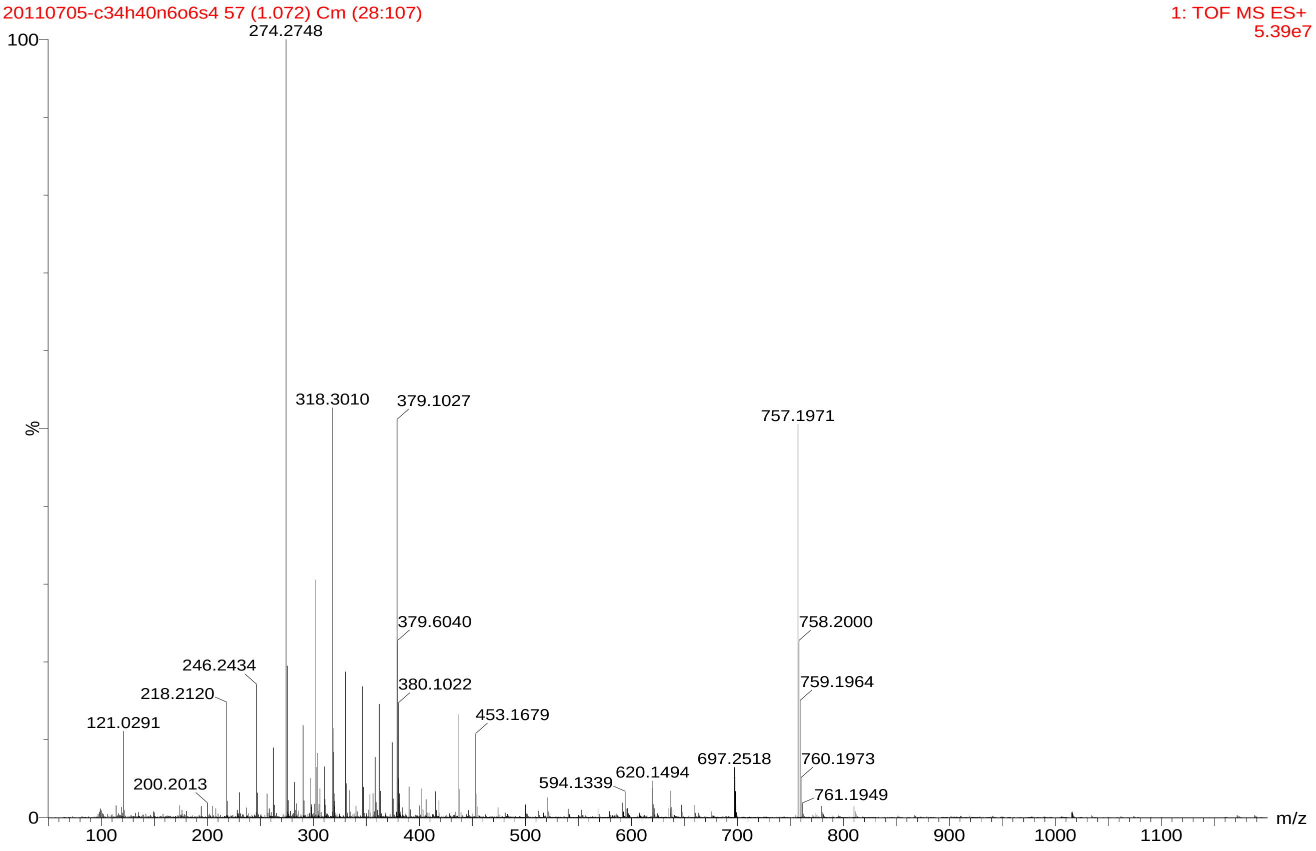

Figure S2. UV spectrum of **1**.

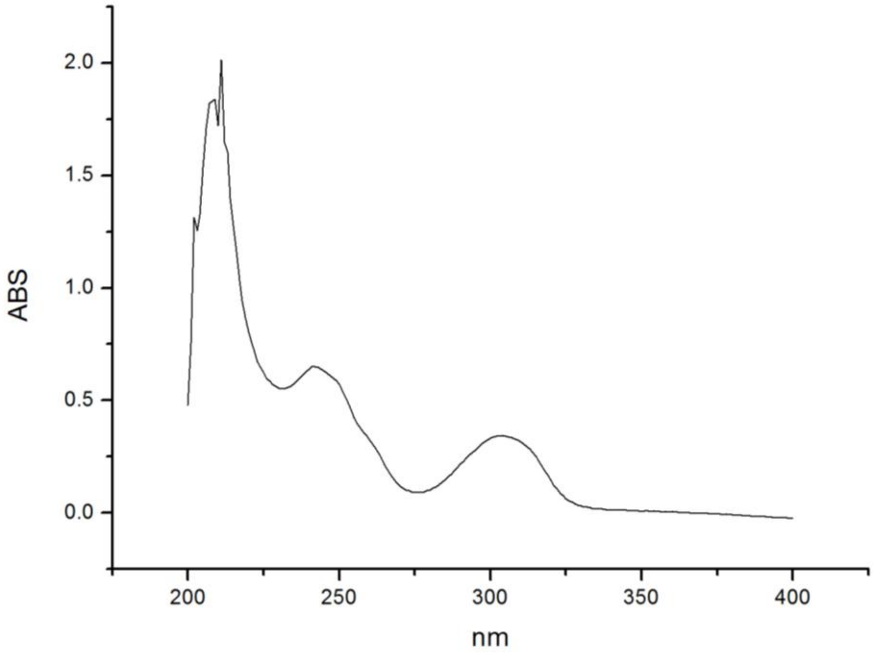

Figure S3. IR spectrum of **1**.

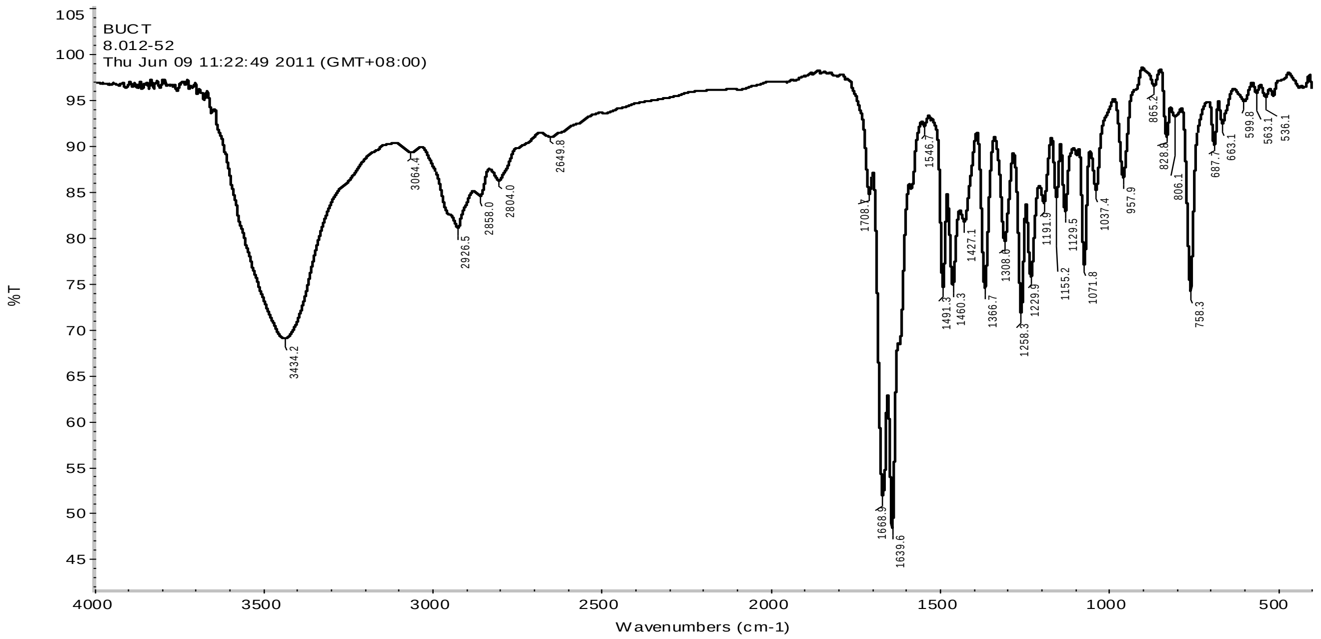

Figure S4. ^{1}H-NMR spectrum (600 MHz, Acetone-d_6) of **1**.

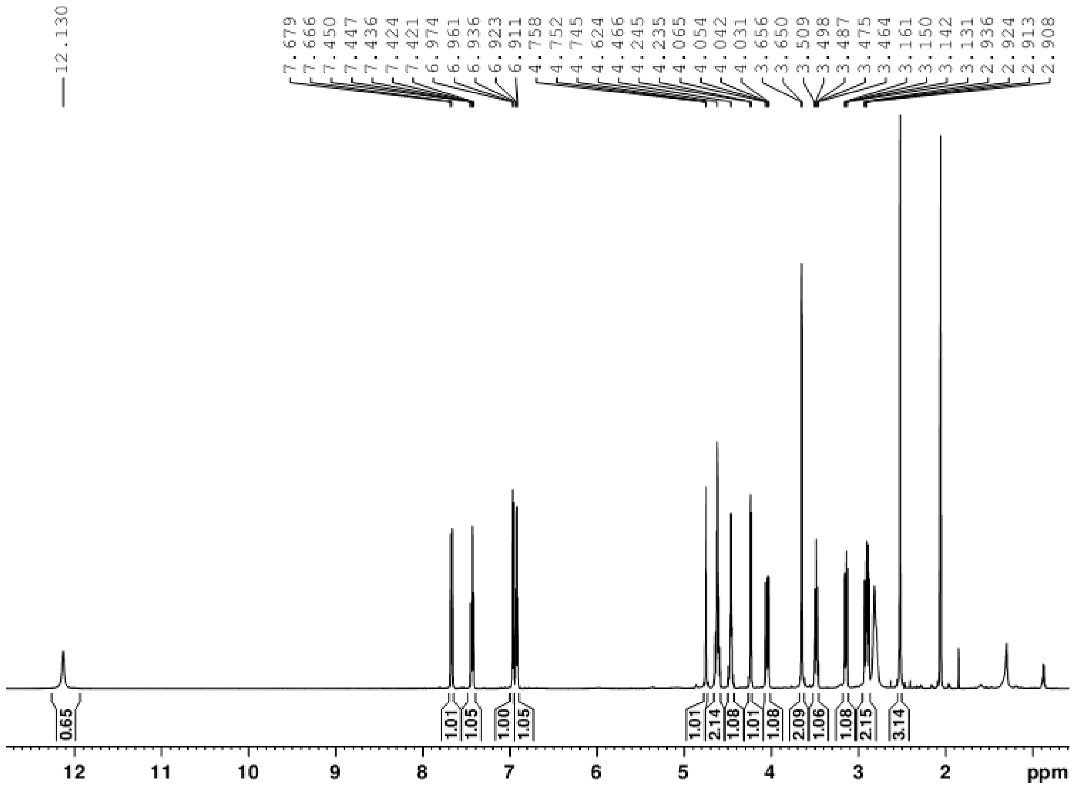

Figure S5. ^{13}C-NMR spectrum (150 MHz, Acetone-d_6) of **1**.

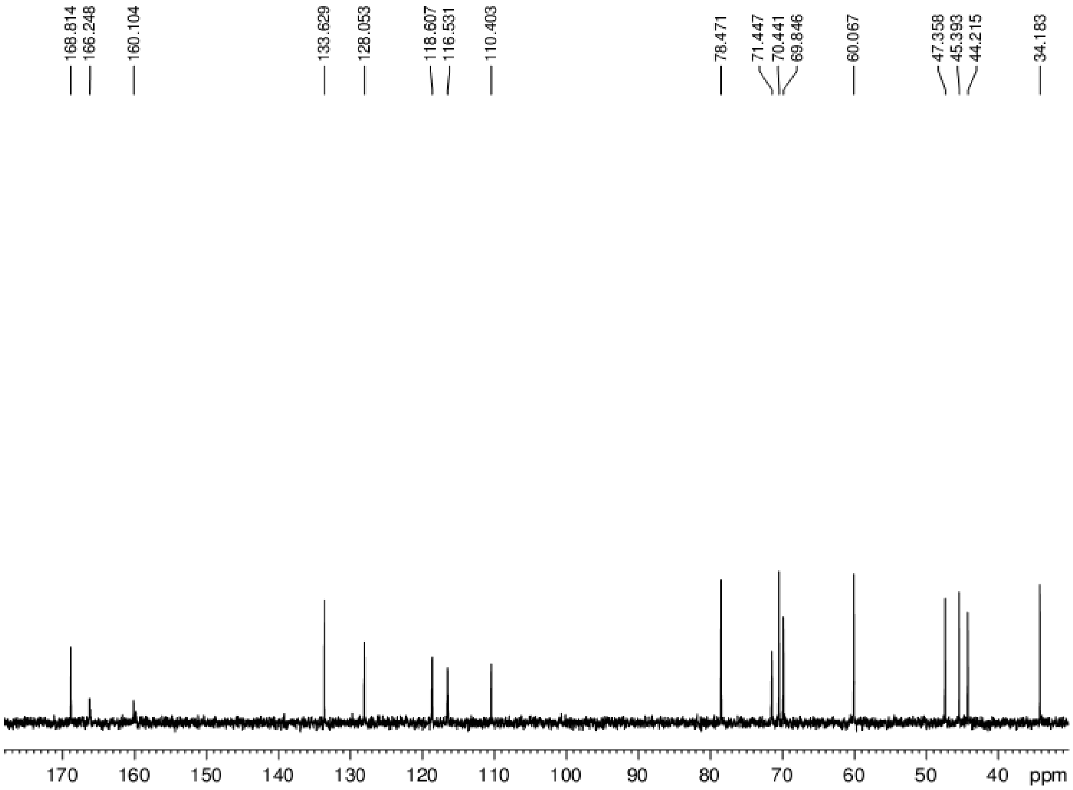

Figure S6. DEPT135 spectrum (150 MHz, Acetone-d_6) of **1**.

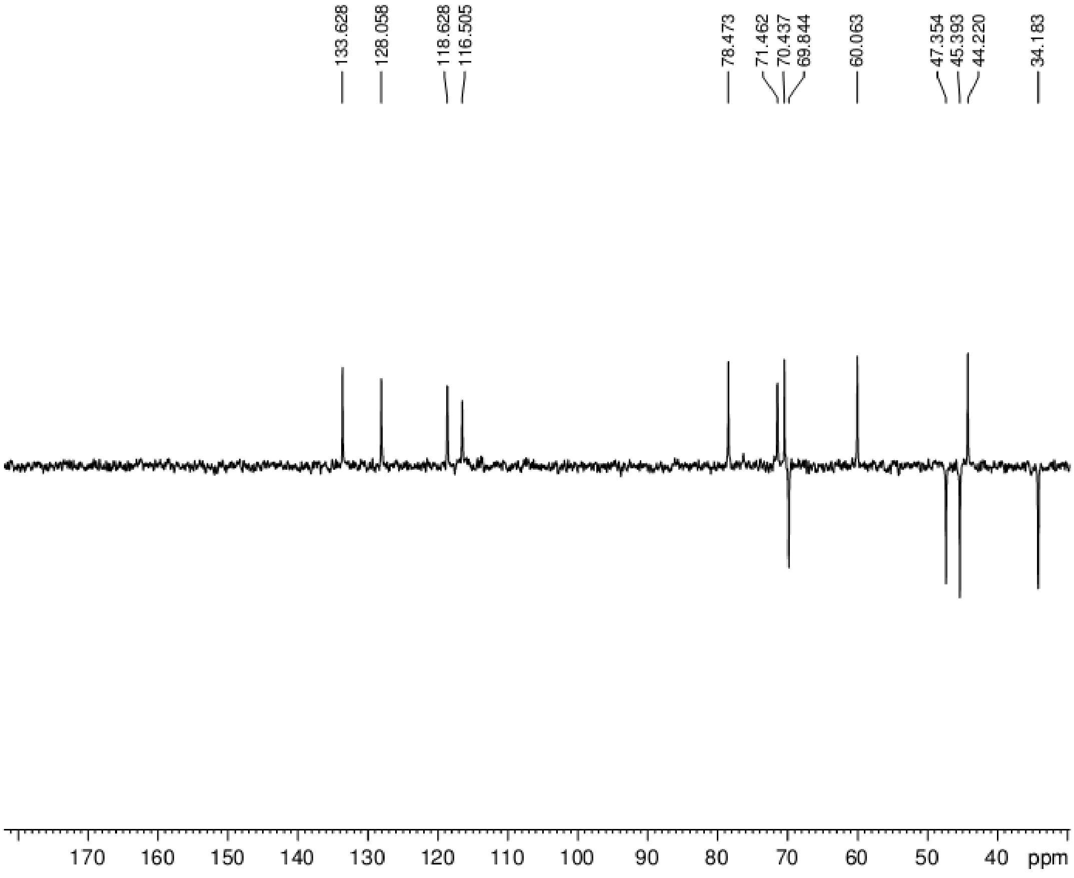

Figure S7. ^{1}H–^{1}H-COSY spectrum (600 × 600 MHz, Acetone-d_6) of **1**.

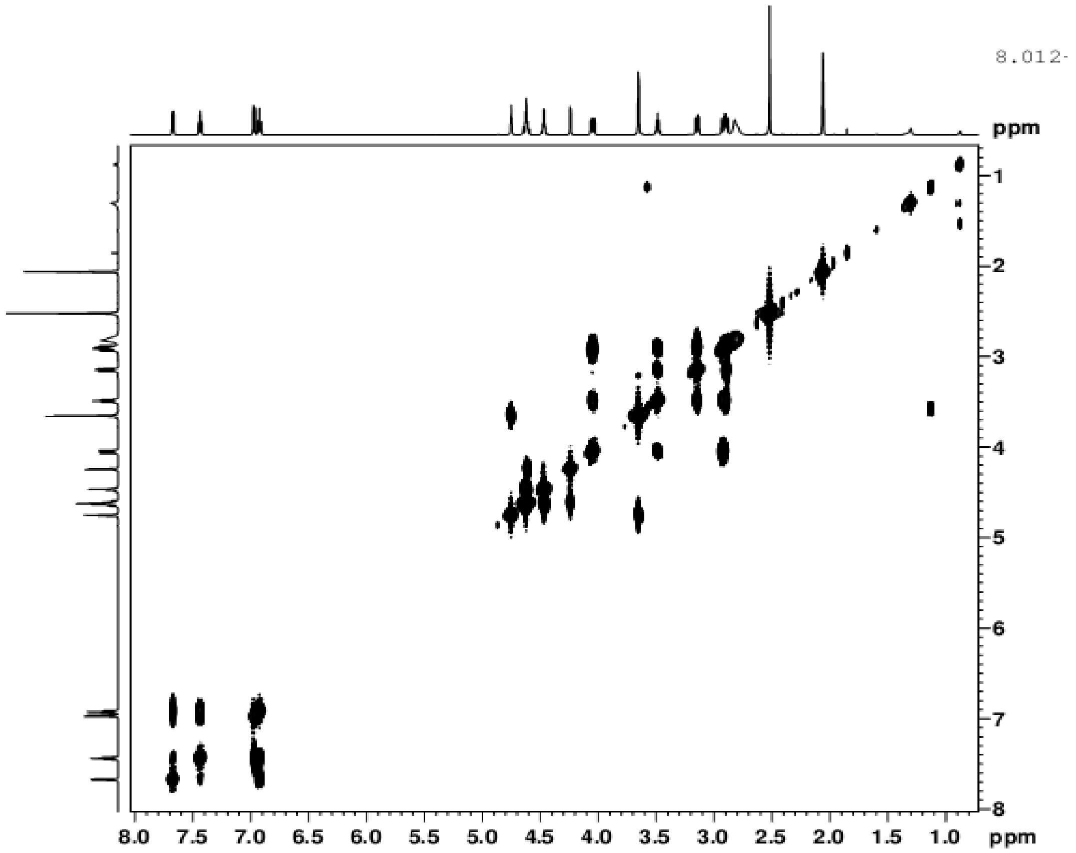

Figure S8. ^{1}H–^{13}C-HSQC spectrum (600 × 150 MHz, Acetone-d_6) of **1**.

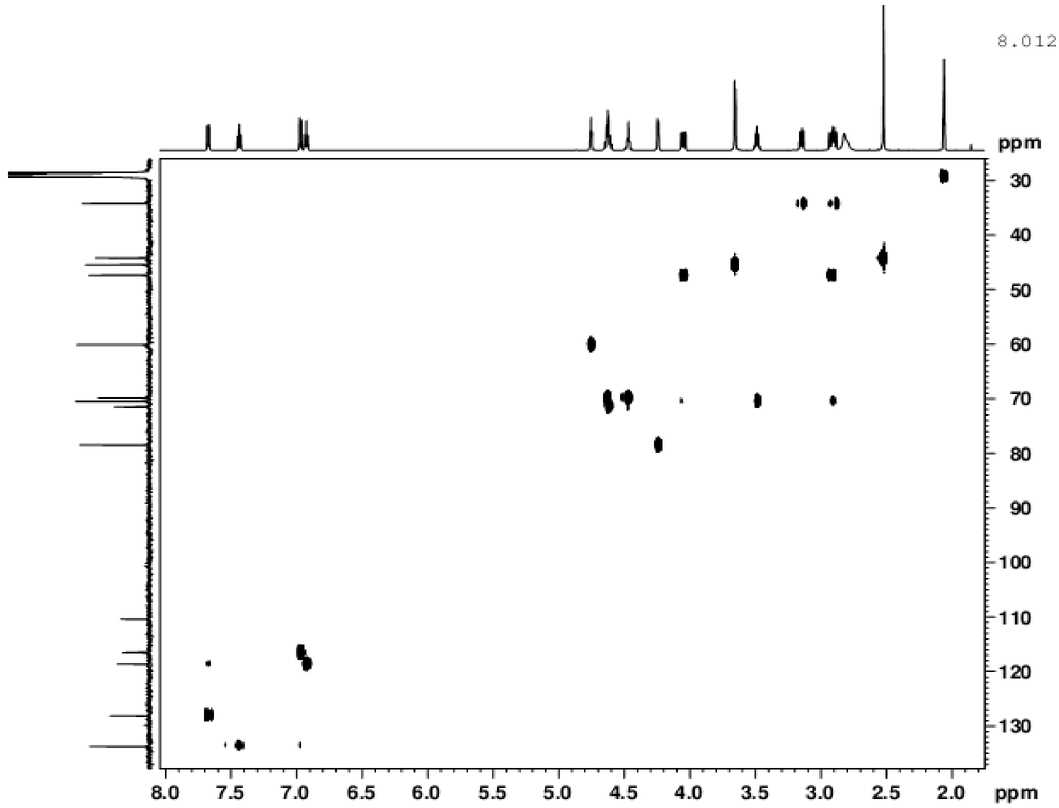

Figure S9. ^{1}H–^{13}C-HMBC spectrum (600 × 150 MHz, Acetone-d_6) of **1**.

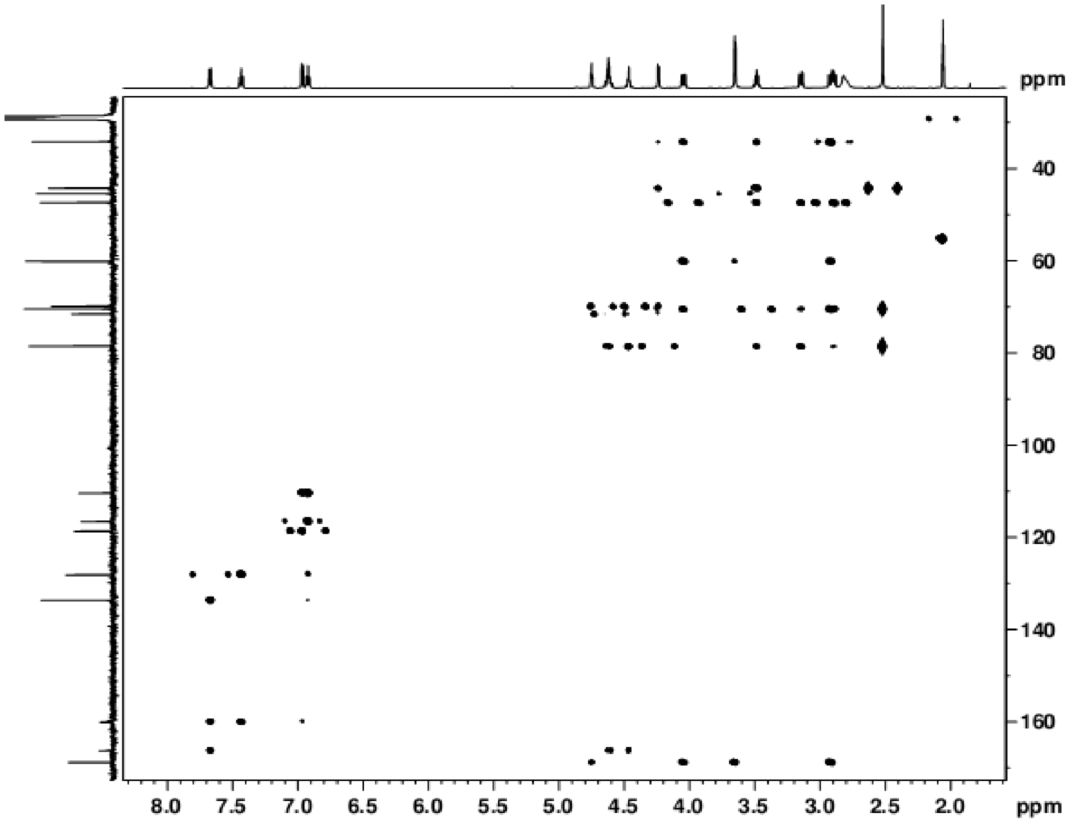

Figure S10. ^{1}H–^{1}H-ROESY spectrum (600 × 600 MHz, Acetone-d_6) of **1**.

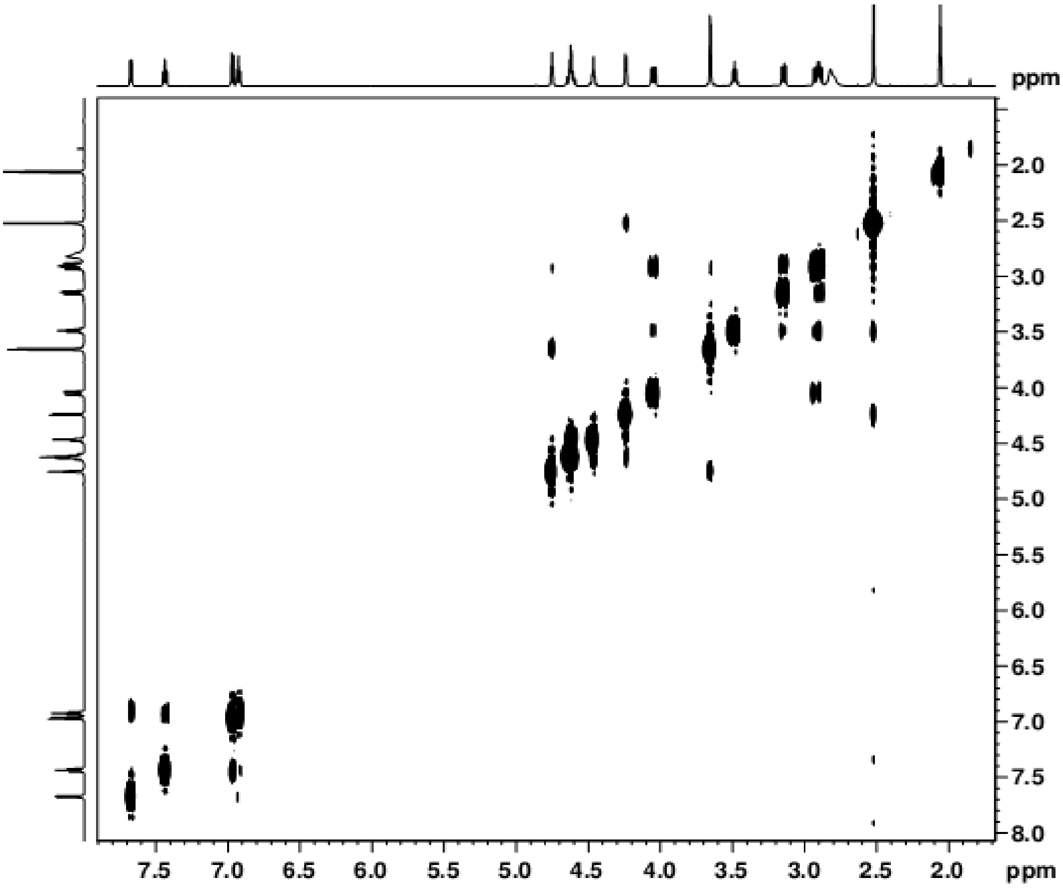

Figure S11. NOE difference spectrum (H-10 irradiated) (600 MHz, Acetone-d_6) of **1**.

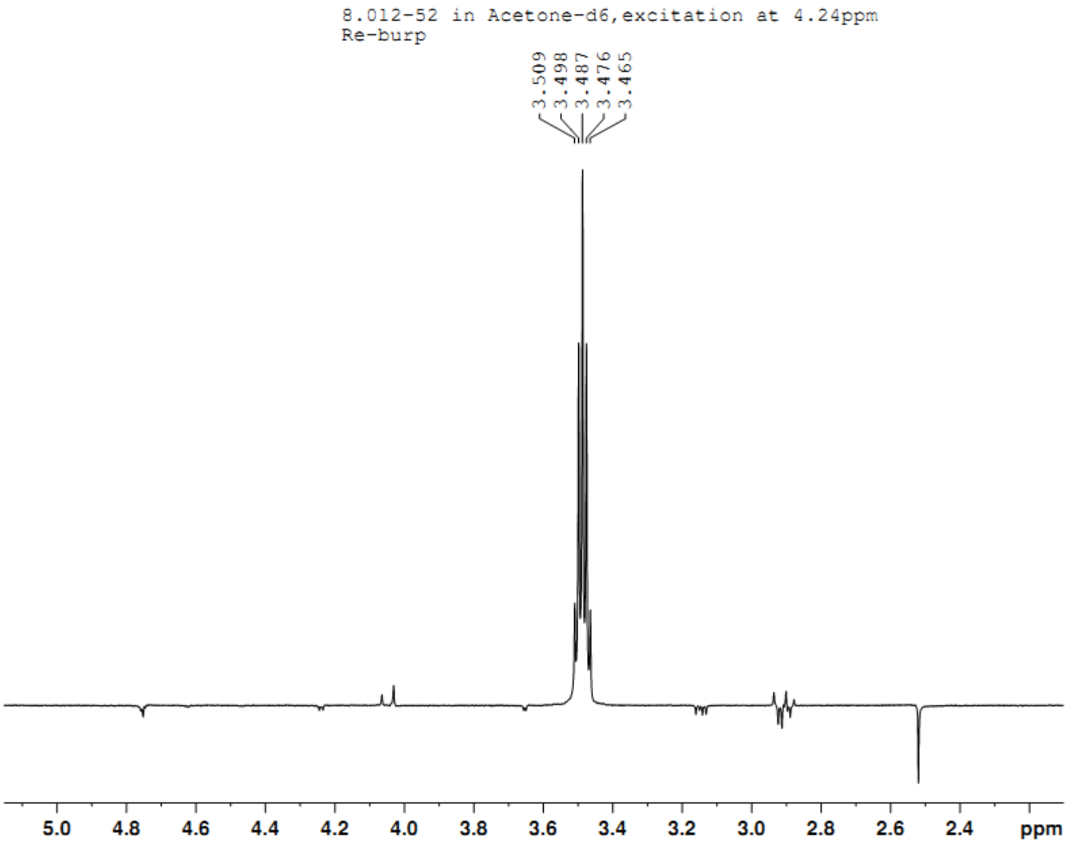

Figure S12. NOE difference spectrum (H-8a irradiated) (600 MHz, Acetone-d_6) of **1**.

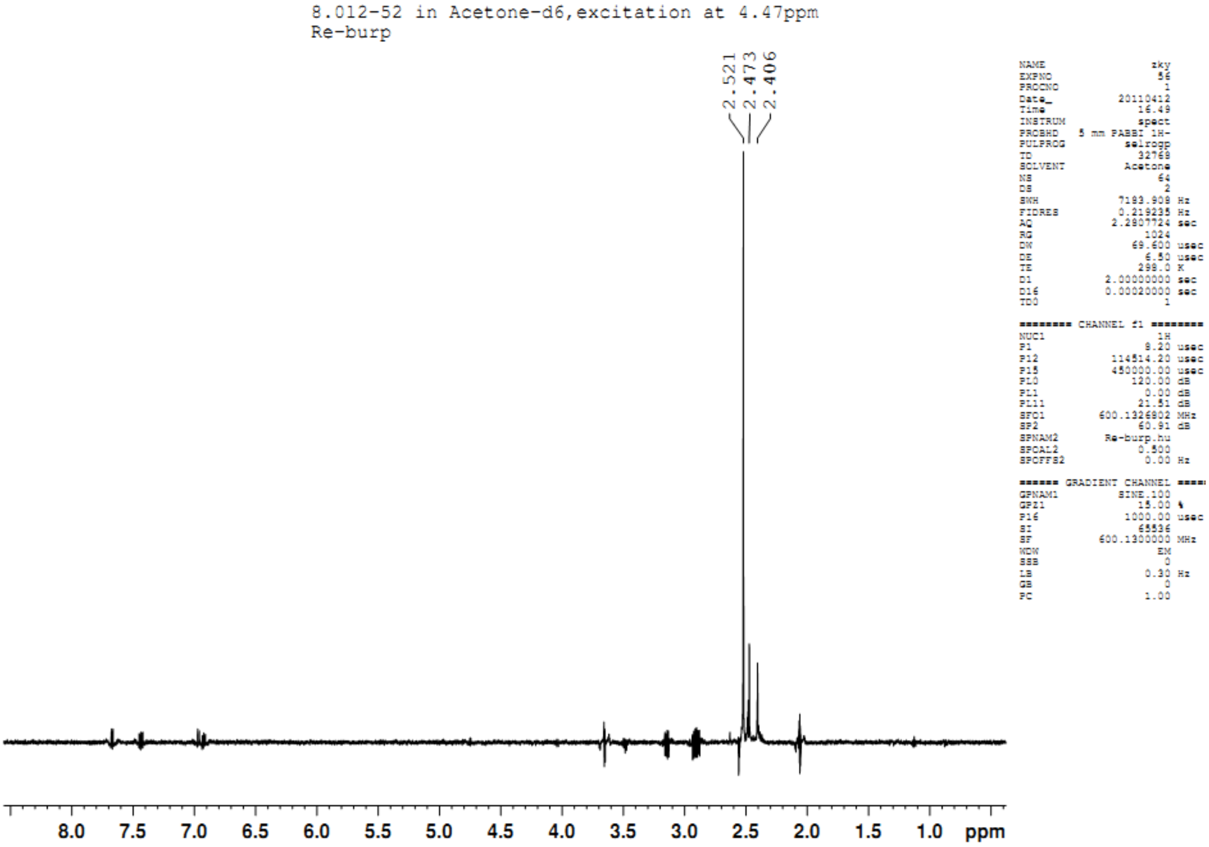

Figure S13. NOE difference spectrum (H-8b irradiated) (600 MHz, Acetone-d_6) of **1.**

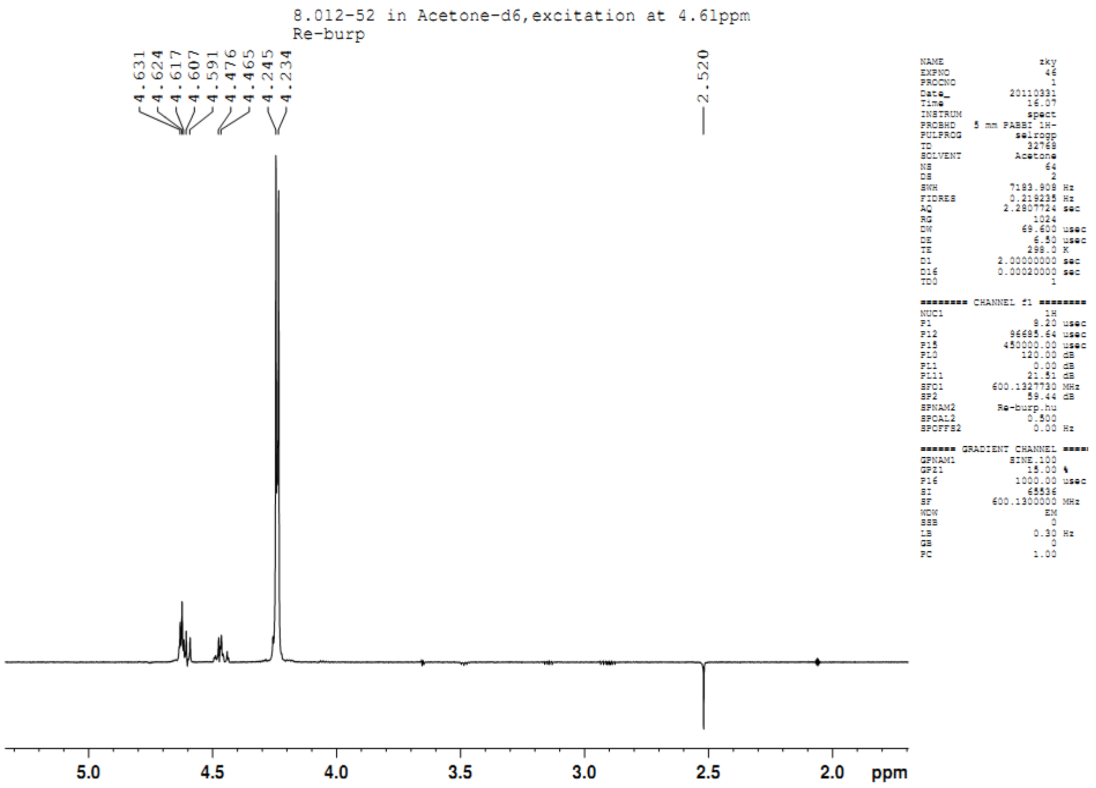

Figure S14. HR-ESI-MS spectrum of **2**.

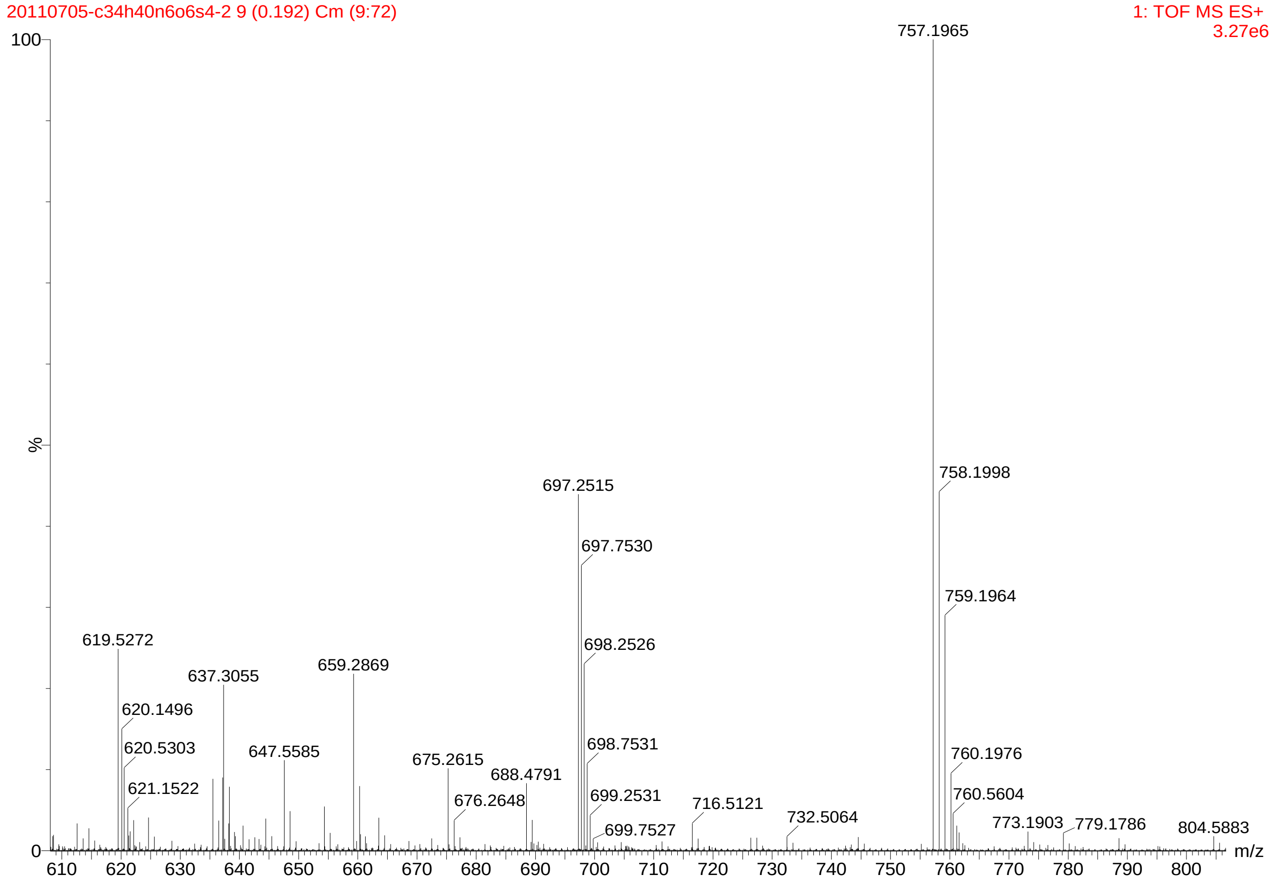

Figure S15. UV spectrum of **2**.

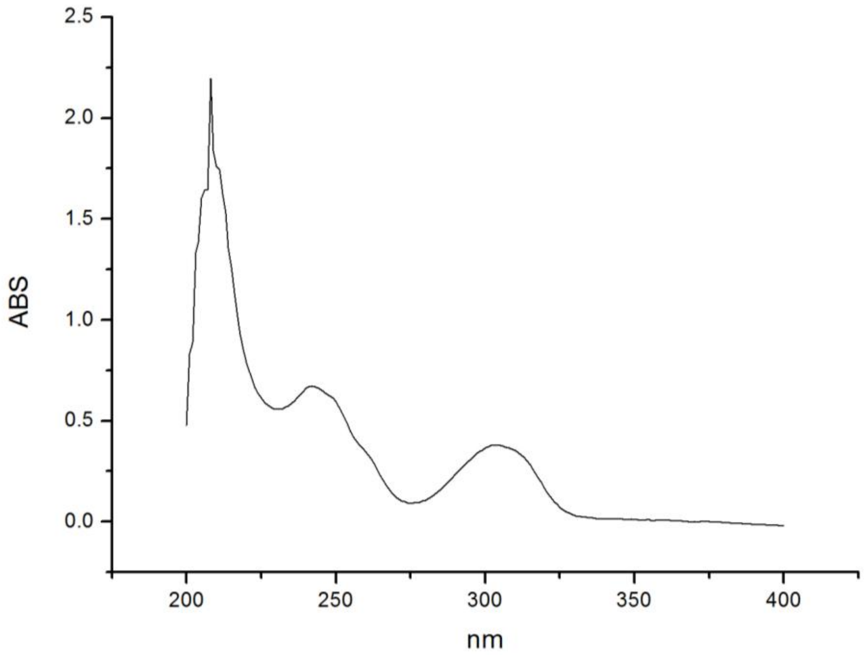

Figure S16. IR spectrum of **2**.

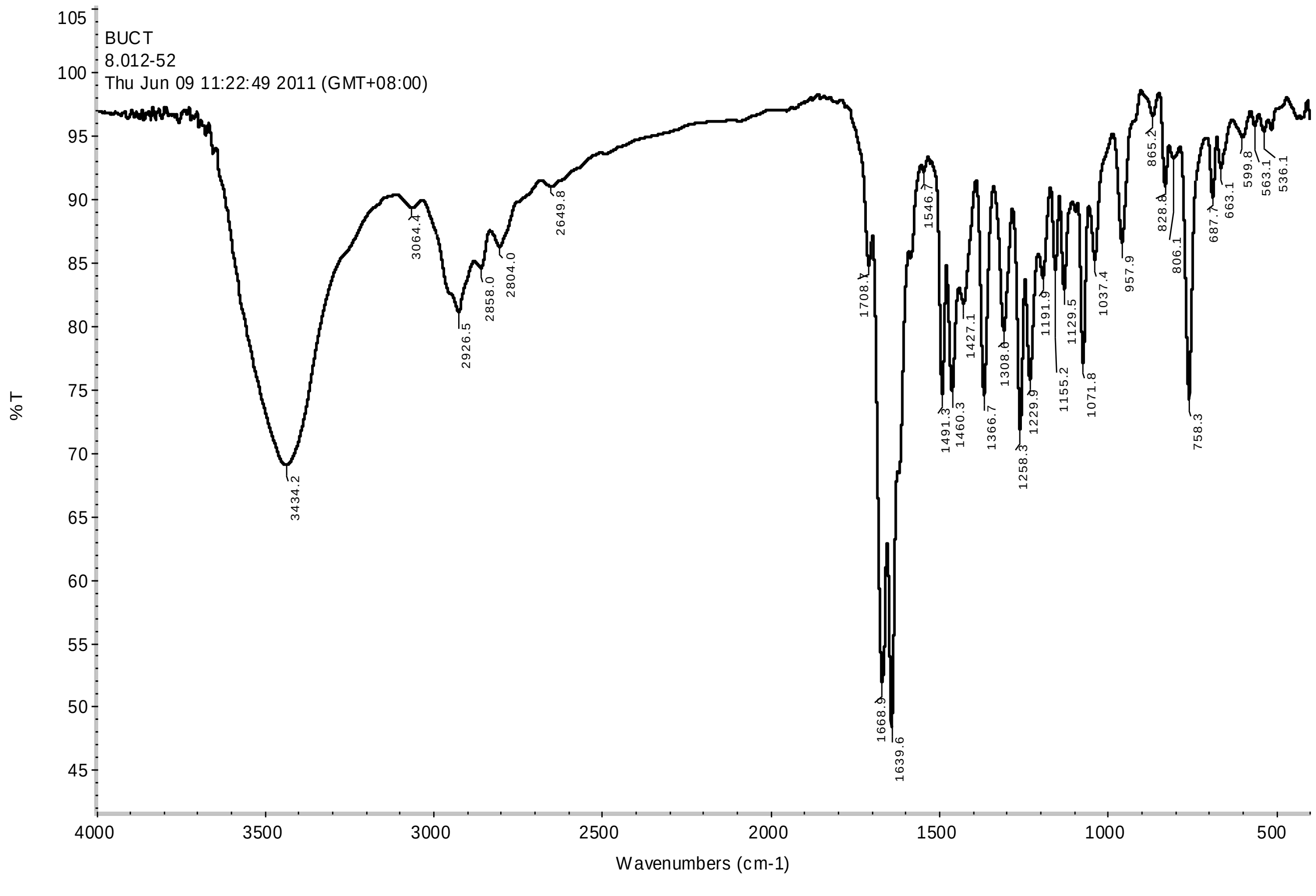

Figure S17. ^{1}H-NMR spectrum (600 MHz, Acetone-d_6) of **2**.

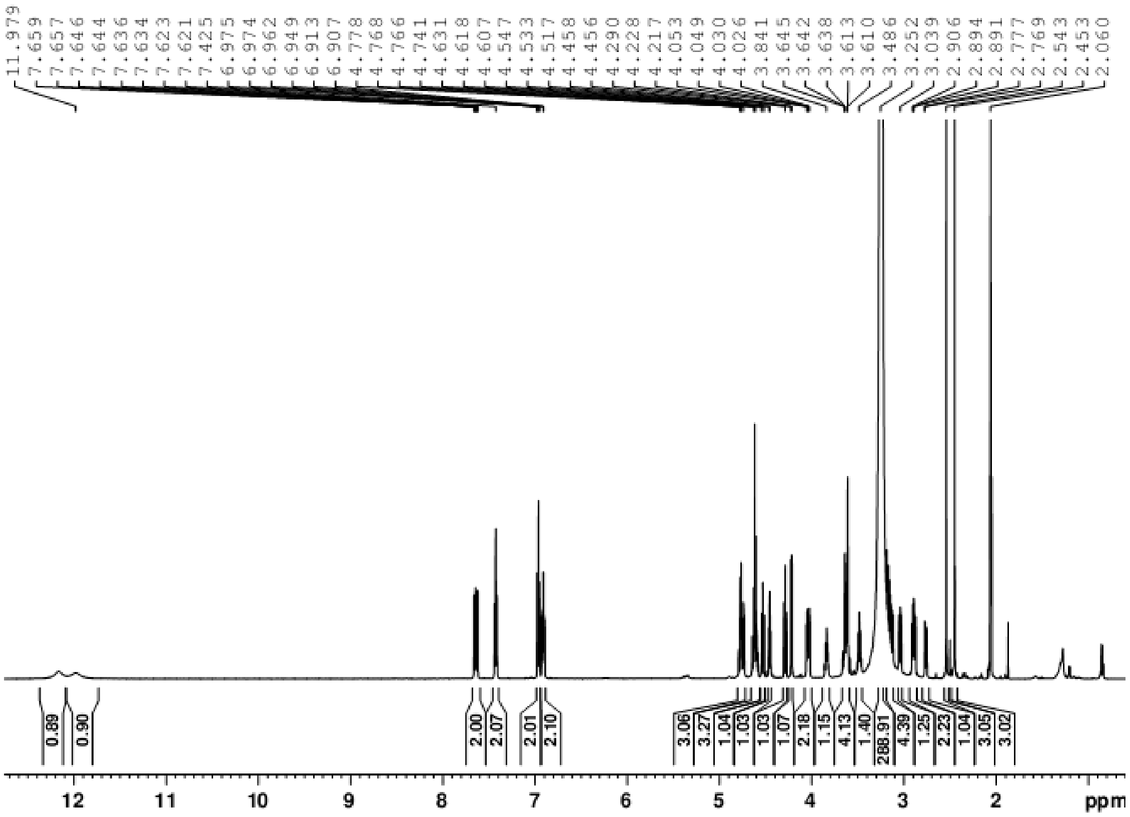

Figure S18. ^{13}C-NMR spectrum (150 MHz, Acetone-d_6) of **2**.

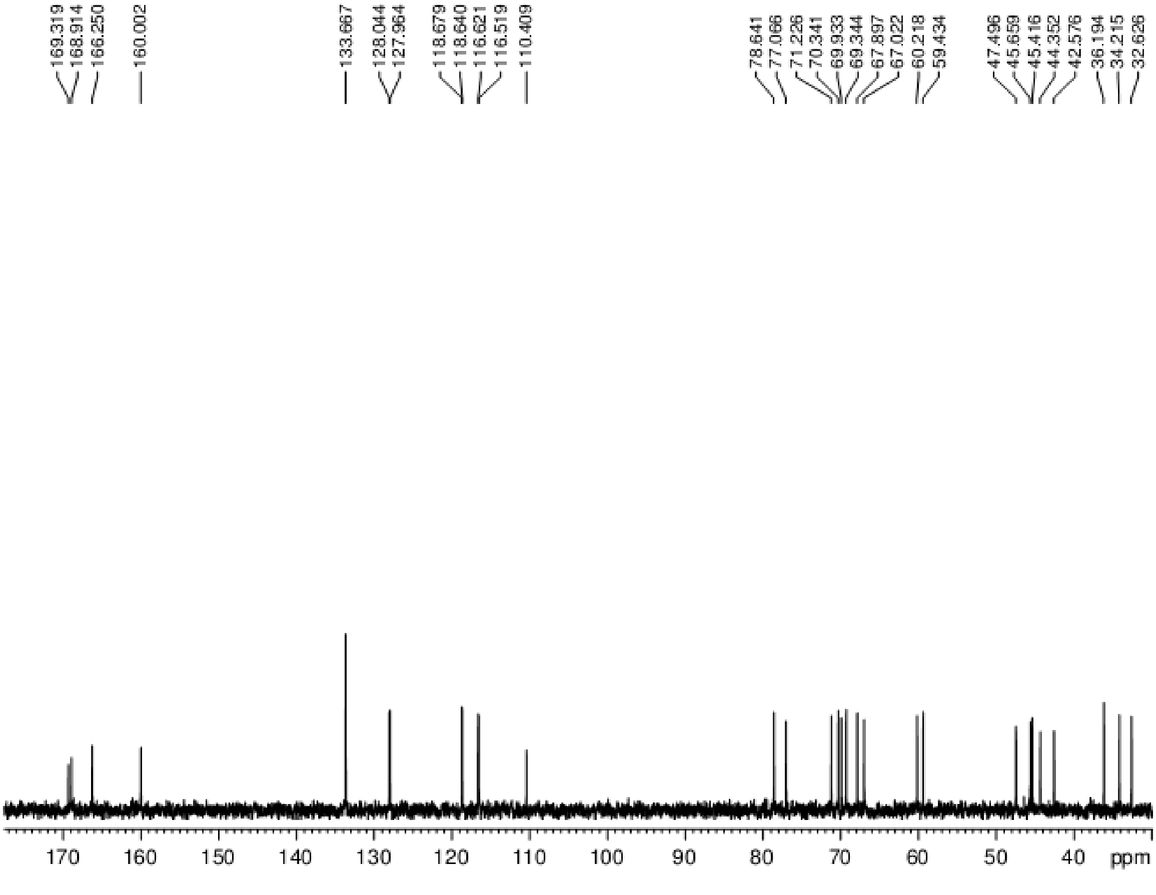

Figure S19. DEPT135 spectrum (150 MHz, Acetone-d_6) of **2**.

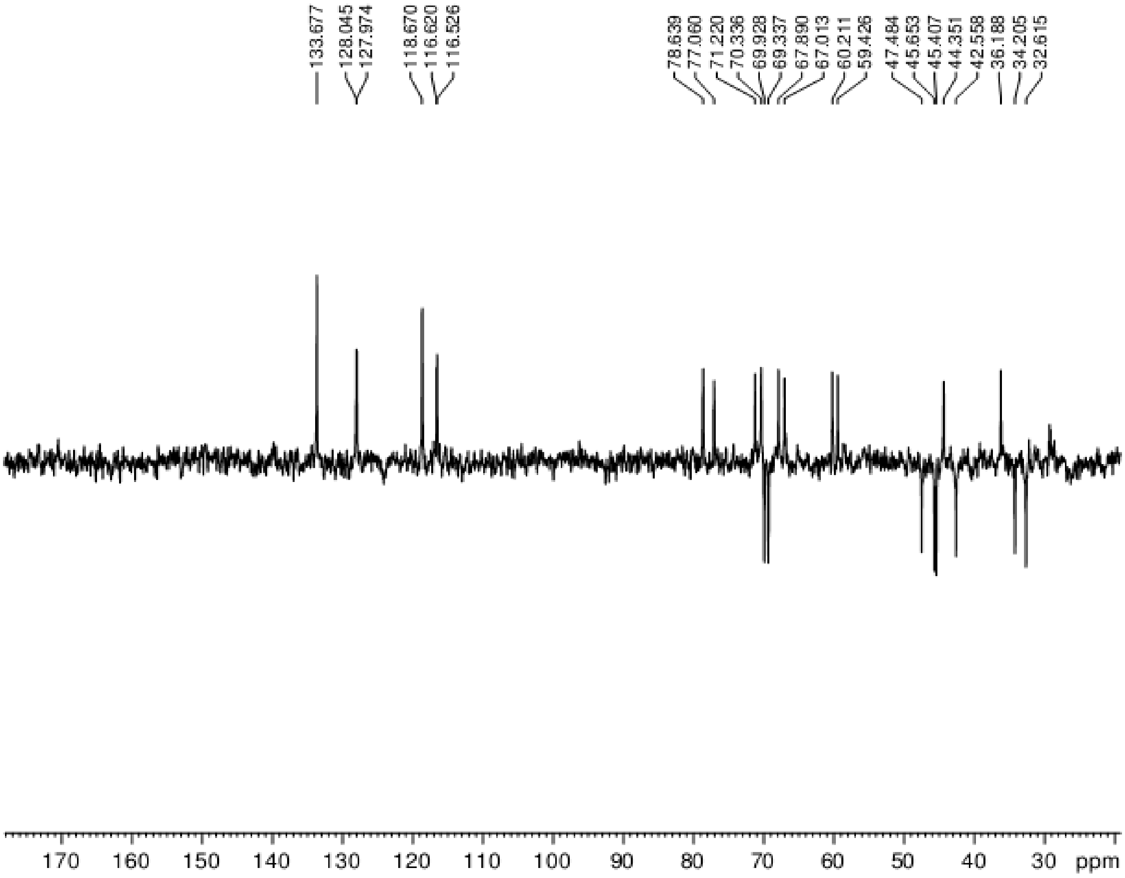

Figure S20. ^{1}H–^{1}H-COSY spectrum (600 × 600 MHz, Acetone-d_6) of **2**.

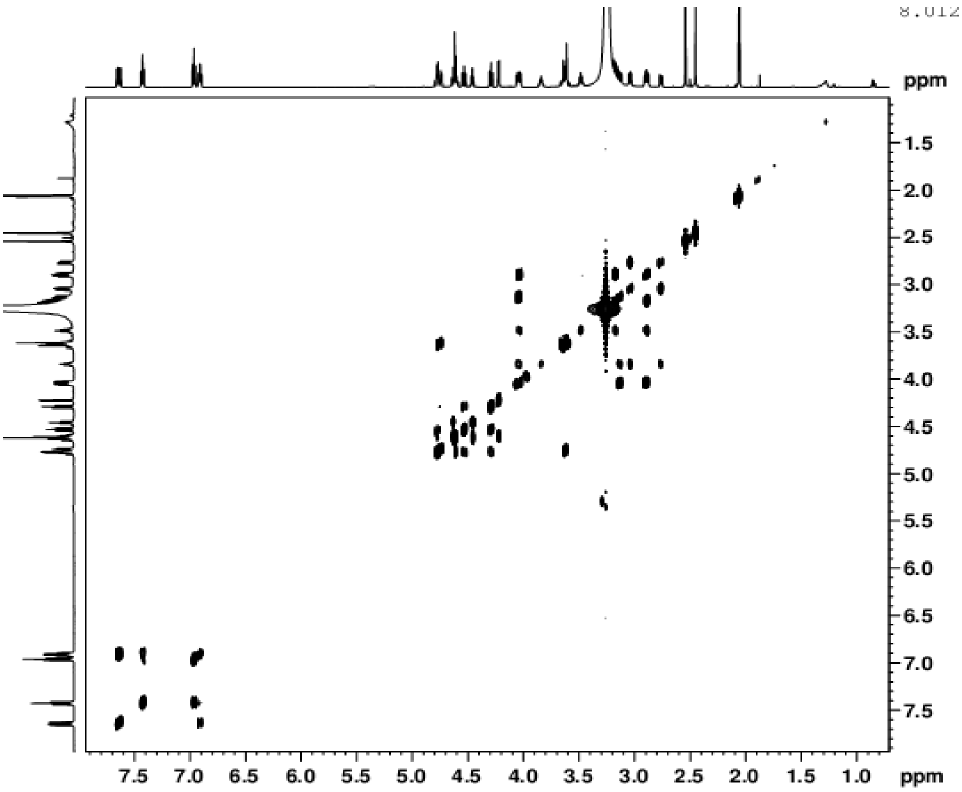

Figure S21. ^{1}H–^{13}C-HSQC spectrum (600 × 150 MHz, Acetone-d_6) of **2**.

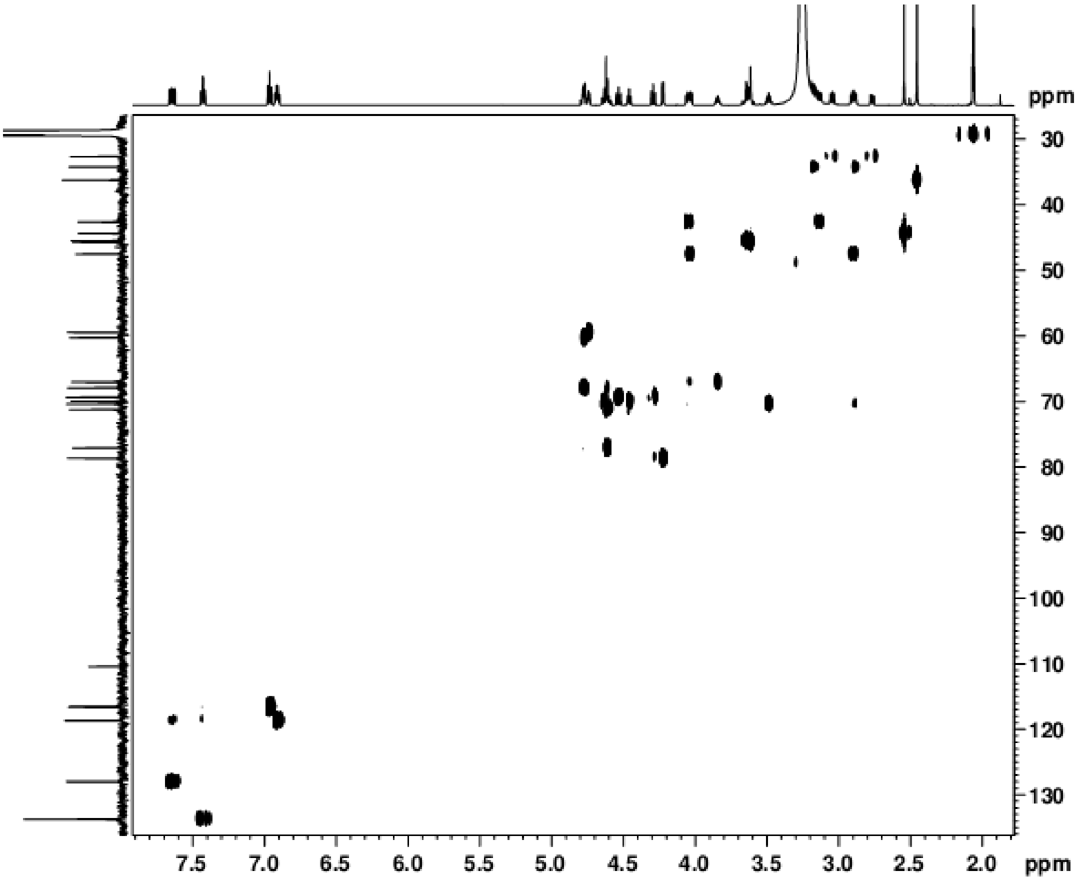

Figure S22. ^{1}H–^{13}C-HMBC spectrum (600 × 150 MHz, Acetone-d_6) of **2**.

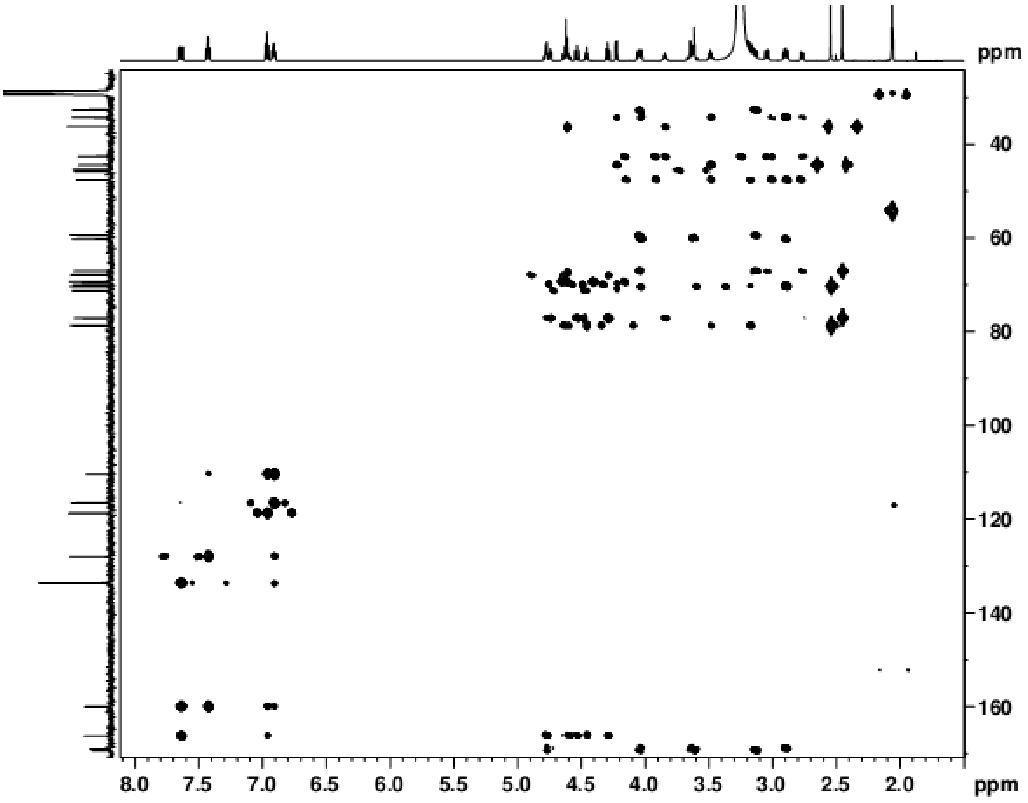

Figure S23. ^{1}H–^{1}H-ROESY spectrum (600 × 600 MHz, Acetone-d_6) of **2**.

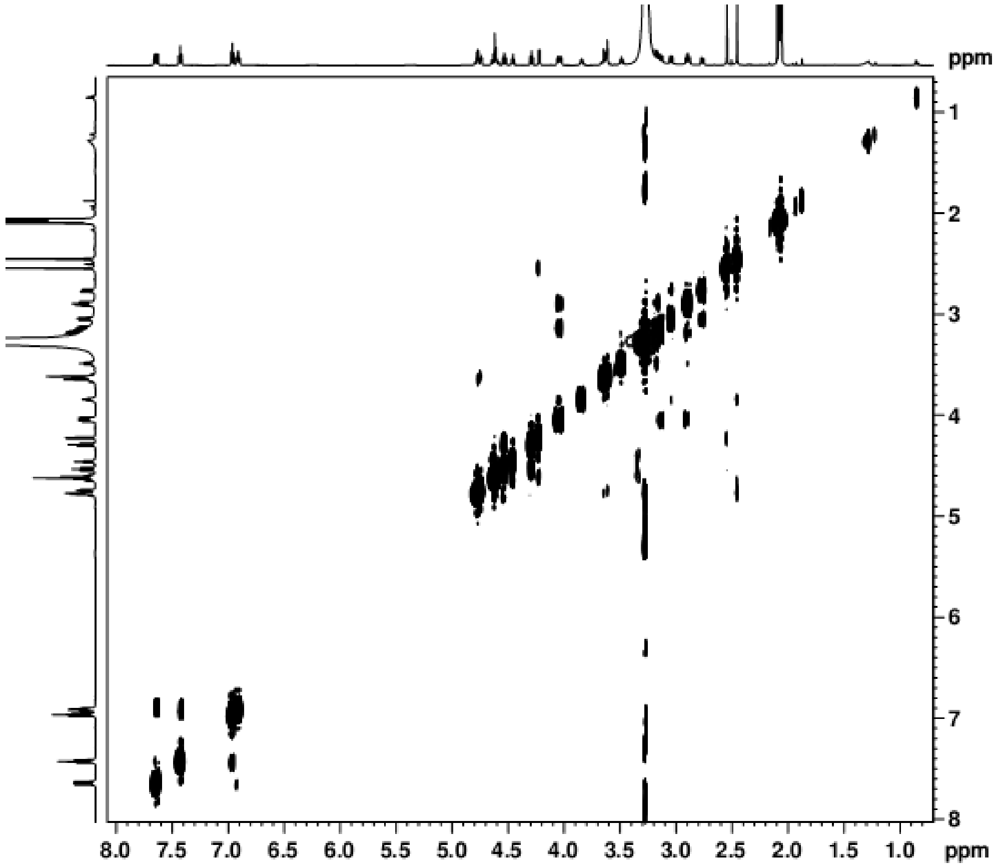

Figure S24. HR-ESI-MS spectrum of **3**.

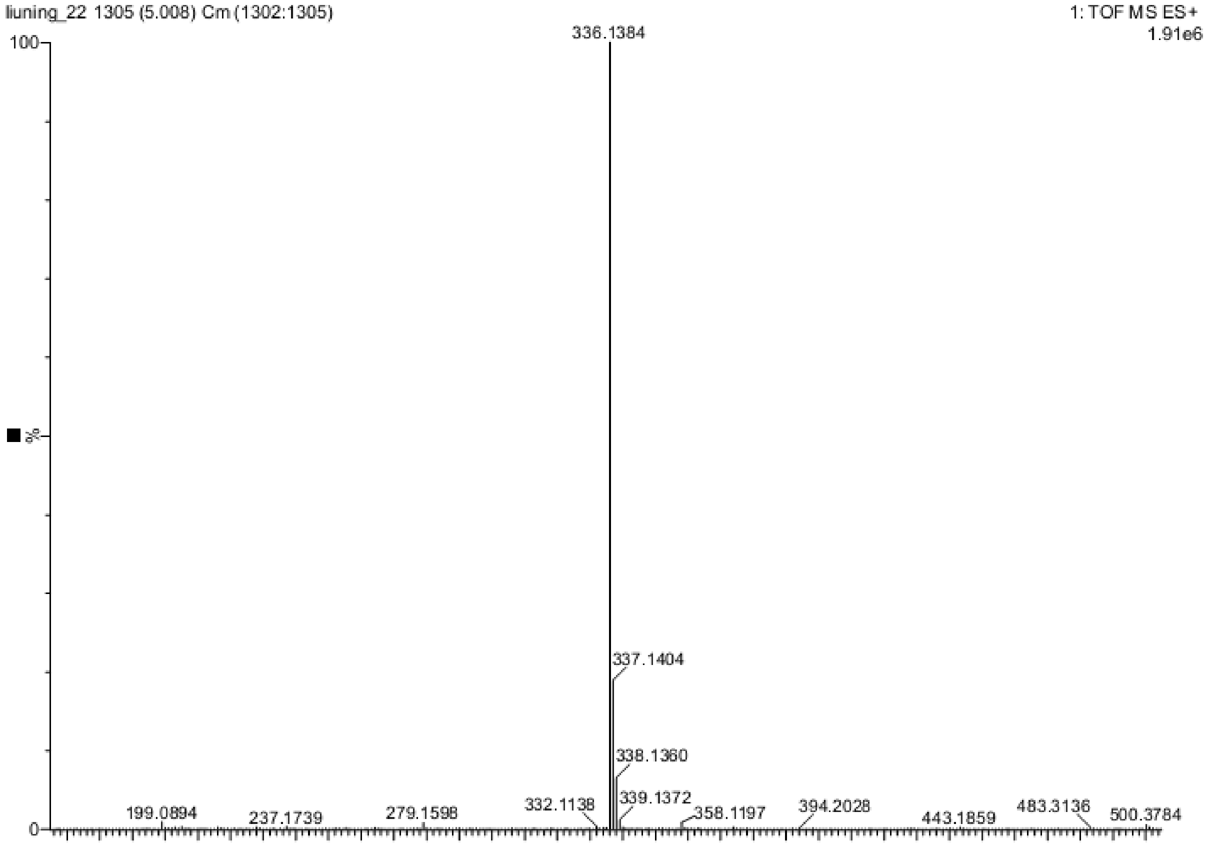

Figure S25. ^{1}H-NMR spectrum (600 MHz, *CD3OD*) of **3**.

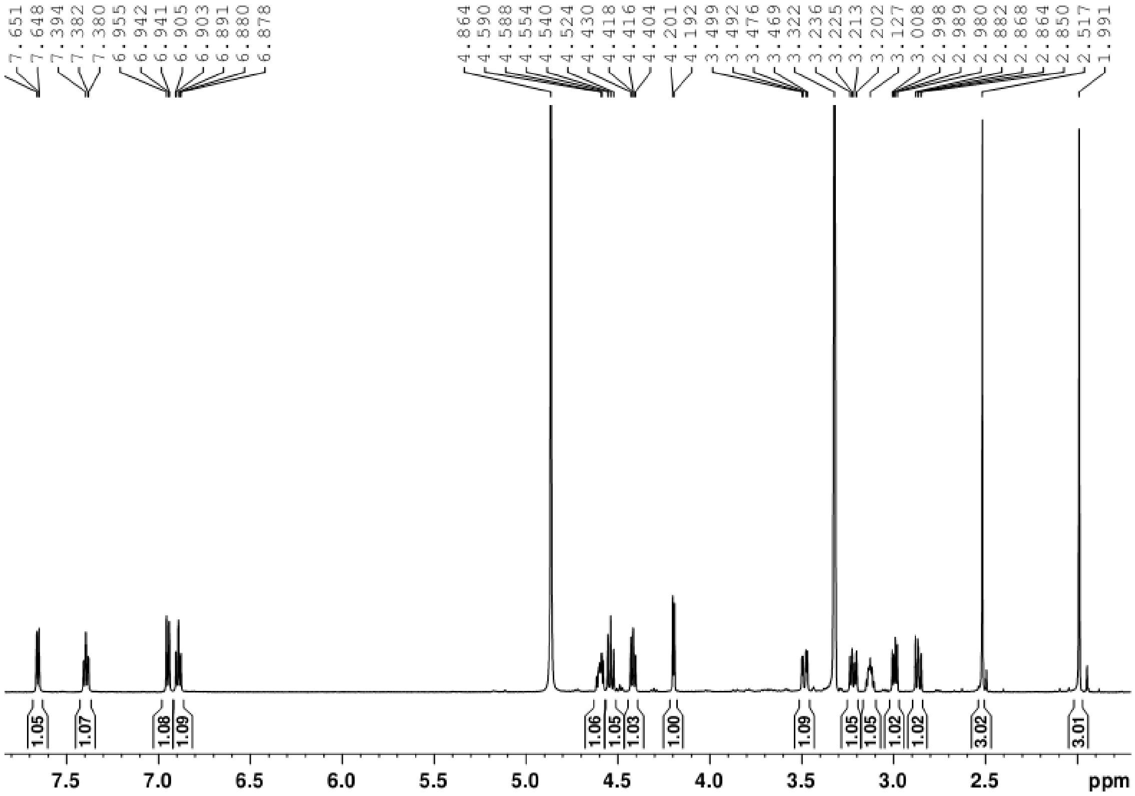

Figure S26. ^{13}C-NMR spectrum (150 MHz, *CD3OD*) of **3**.

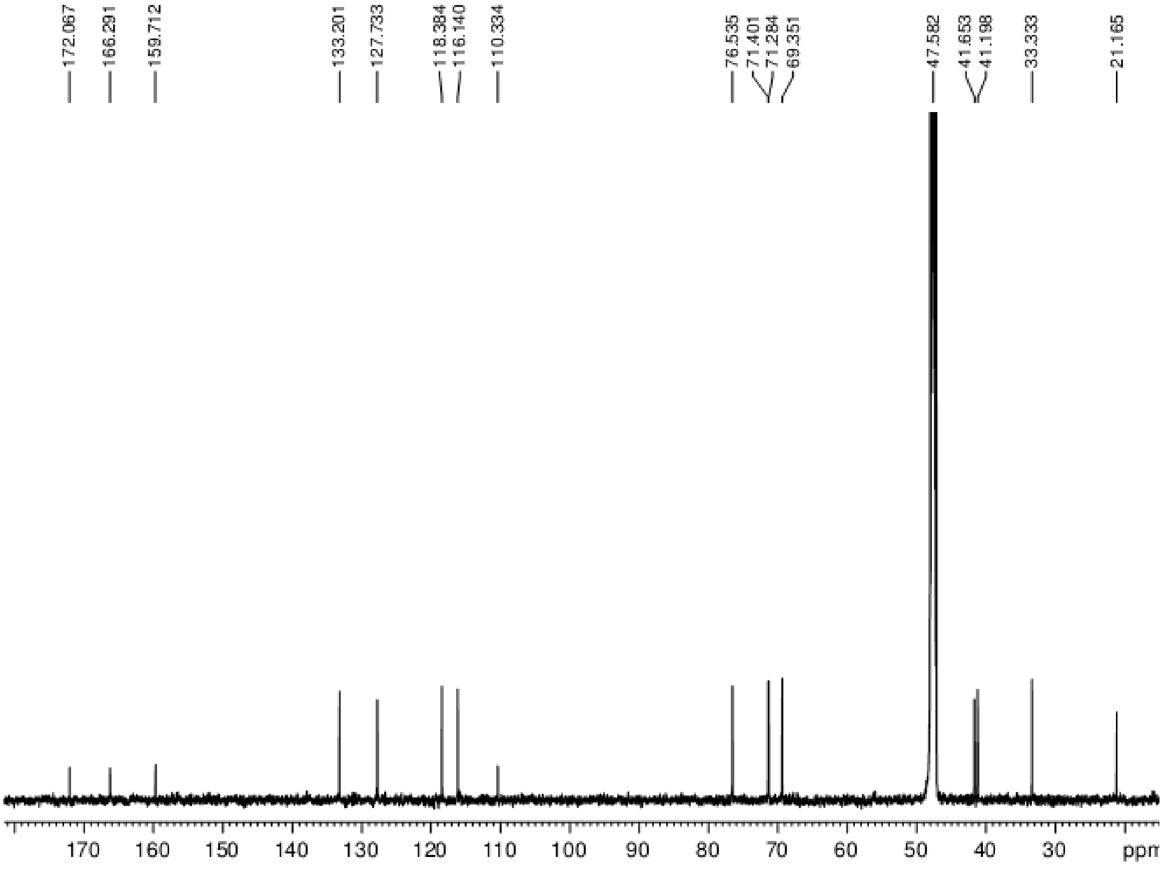

Figure S27. HR-ESI-MS spectrum of **4**.

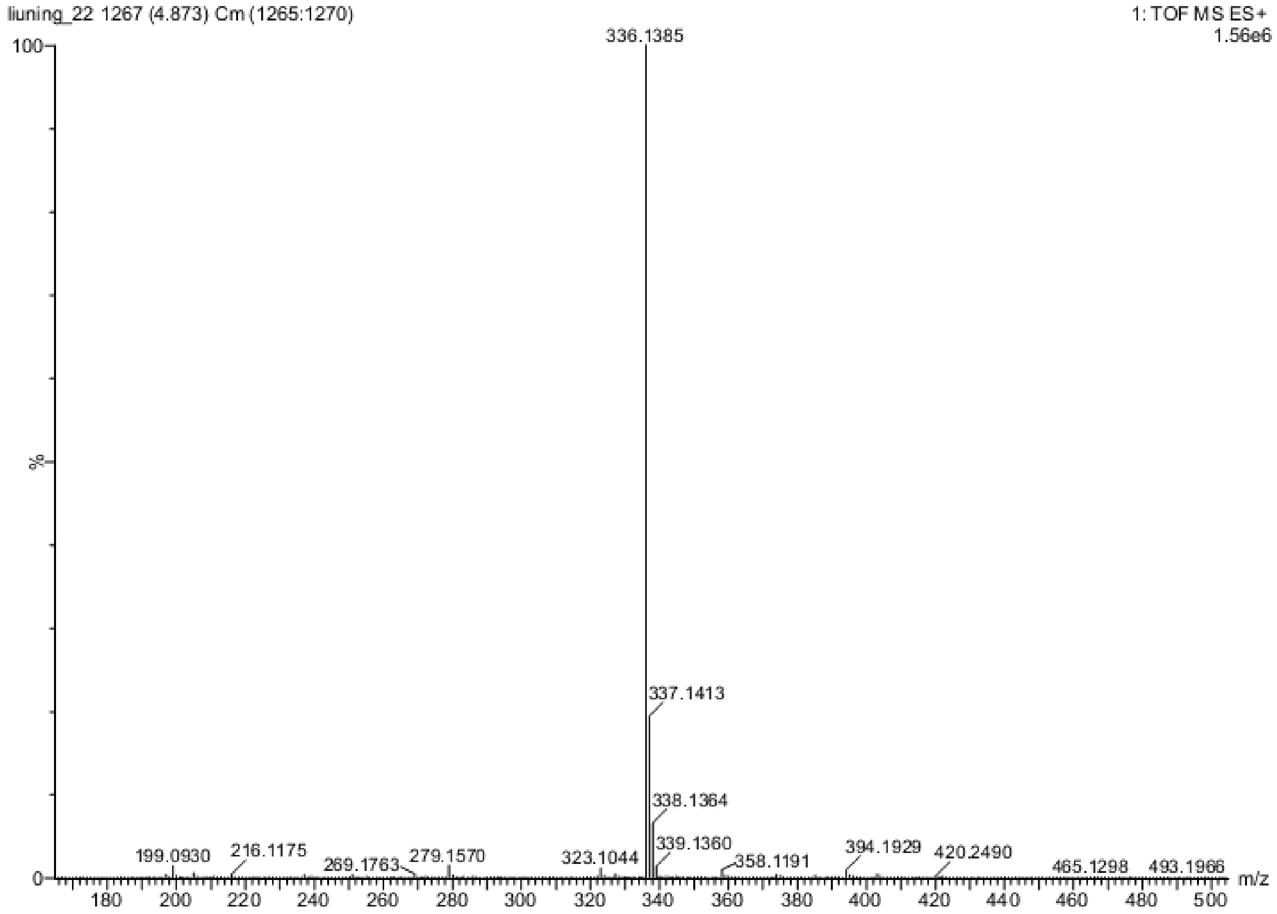

Figure S28. ^{1}H-NMR spectrum (600 MHz, *CD_3OD*) of **4**.

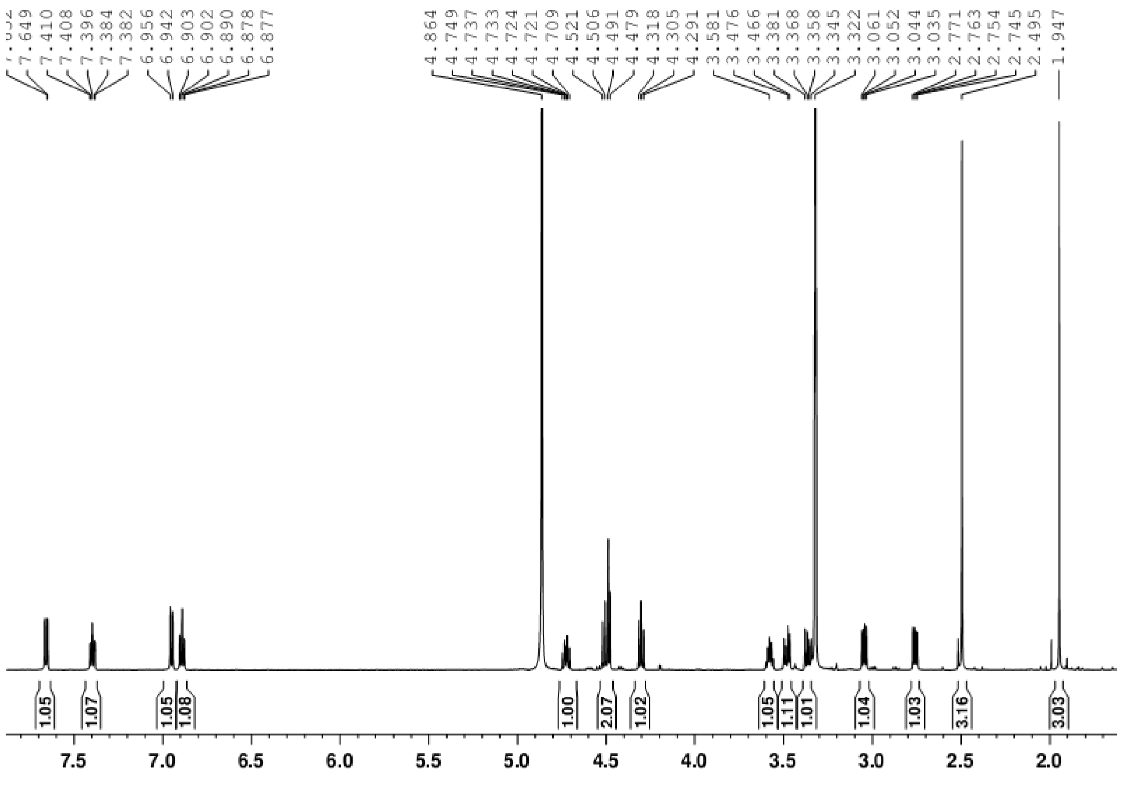

Figure S29. ^{13}C-NMR spectrum (150 MHz, CD_3OD) of **4**.

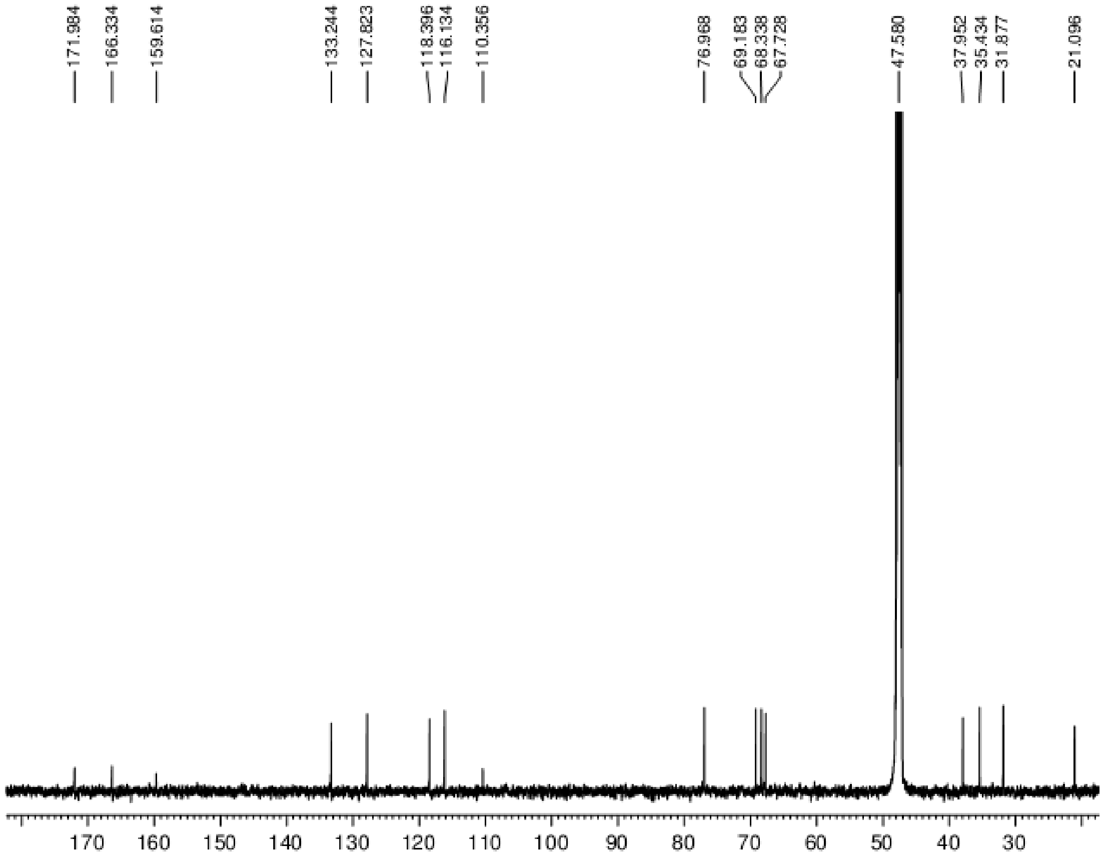

Figure S30. HR-ESI-MS spectrum of **5**.

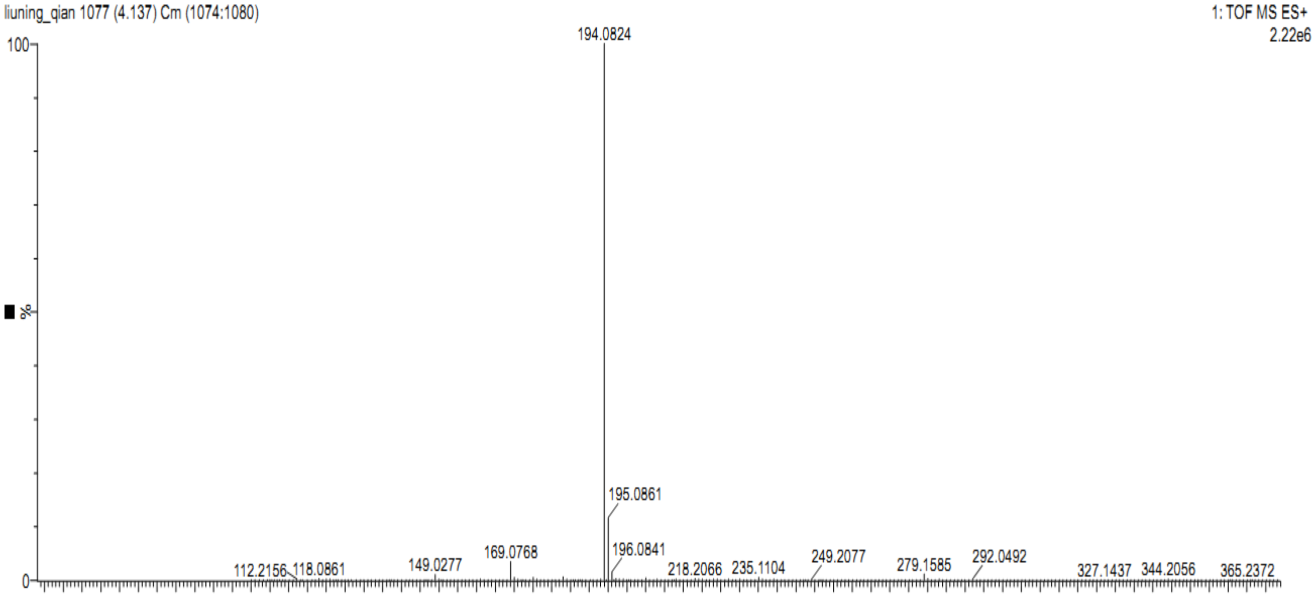

Figure S31. ^{1}H-NMR spectrum (400 MHz, Acetone-d_6) of **5**.

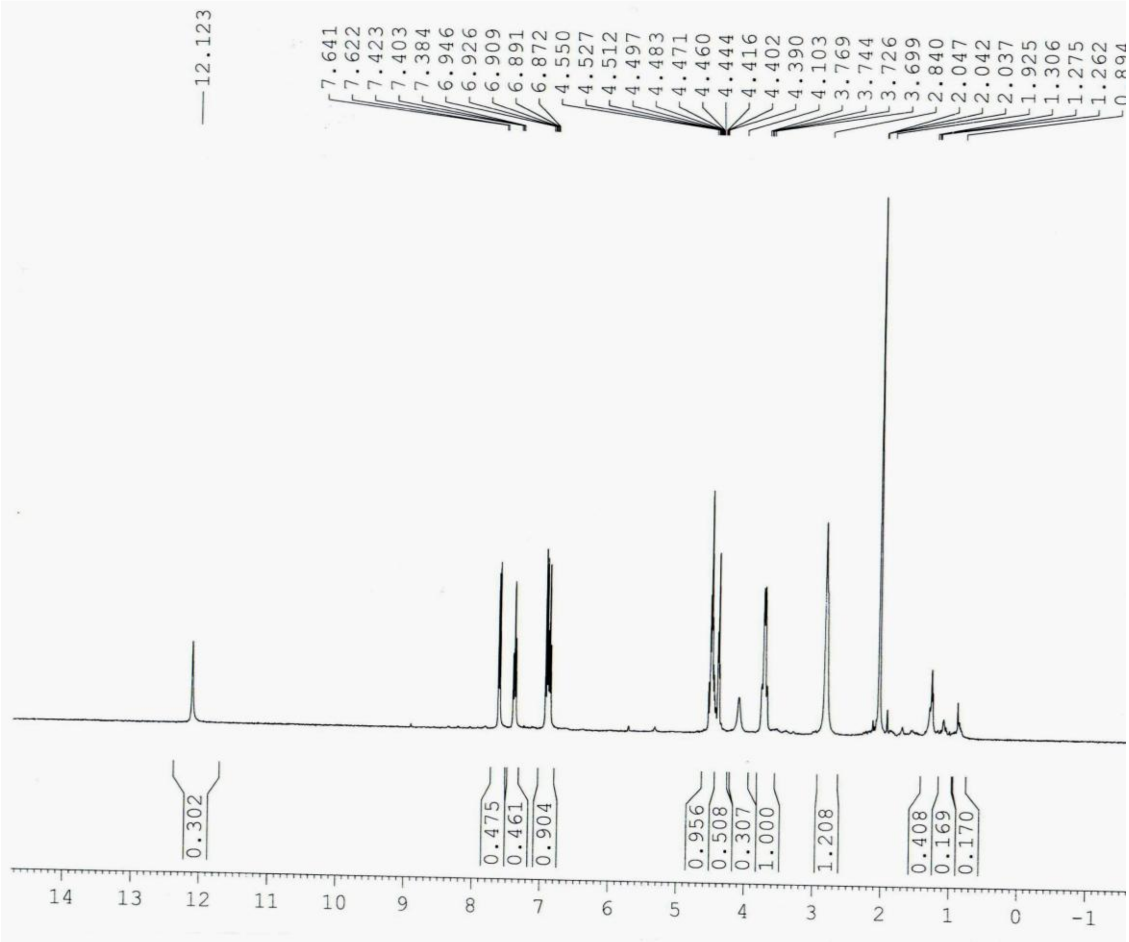

Supplement: Supplementary File 1 — Supplementary (PDF, 807 KB) [file marinedrugs-11-01524-s001.pdf]
